# Which commercial Chinese polyherbal preparations combined with ACEI/ARB is effective and safe for IgA nephropathy? A systematic review and network meta-analysis

**DOI:** 10.3389/fphar.2026.1755995

**Published:** 2026-06-24

**Authors:** Li Zheng, Xiaotong Gu, Lin Zhang, Chumeng Yang, Xuelin Sun, Weina Zhang, Songtao Yang

**Affiliations:** 1 Department of Pharmacy, Beijing Genertec Aerospace Hospital, Beijing, China; 2 Department of Pharmacy, Beijing Hospital, National Center of Gerontology, Institute of Geriatric Medicine, Chinese Academy of Medical Sciences, Beijing, China; 3 Department of Nephrology, Beijing Genertec Aerospace Hospital, Beijing, China

**Keywords:** ACEI/ARB, commercial Chinese polyherbal preparations, GRADE, immunoglobulin a nephropathy, network meta-analysis

## Abstract

**Background:**

This systematic review and network meta-analysis (NMA) evaluates and compares the efficacy and safety of six commercial Chinese polyherbal preparations (Bailing Capsule (BL), Huangkui Capsule (HK), Tripterygium Glycosides (TG), Jinshuibao Capsule (JSB), Huobahuagen Tablet (HBHG), and Shenyan Kangfu Tablet (SYKF)) combined with angiotensin-converting enzyme inhibitors or angiotensin II receptor blockers (ACEI/ARB) for treating Immunoglobulin A nephropathy (IgAN), offering updated evidence to support clinical decision-making.

**Methods:**

We conducted a systematic literature search in PubMed, Embase, the Cochrane Library, ClinicalTrials.gov, SinoMed, China National Knowledge Infrastructure (CNKI), Wanfang Data Knowledge Service Platform, and VIP Database for Technology Periodicals (up to 20 October 2025) for randomized controlled trials (RCTs) that enrolled patients with IgAN and compared the combination of six commercial Chinese polyherbal preparations with ACEI/ARB against ACEI/ARB therapy alone. To synthesise the evidence from the network of treatments, we performed frequentist network meta-analyses and subsequently assessed the certainty of evidence through the systematic application of the GRADE (Grading of Recommendations Assessment, Development and Evaluation) approach. The study is registered in the PROSPERO database (CRD420251175045).

**Results:**

A total of 70 RCTs involving 4855 patients were included in this systematic review and NMA. The overall risk of bias was high among the included studies. Compared with ACEI/ARB alone, BL combined with ACEI/ARB probably improves overall response rate (OR: 3.27, 95% CI: 2.21 to 4.84; moderate certainty) and reduces 24‐h urine protein quantification (MD: −0.83 g, 95% CI: −1.21 to −0.44; moderate certainty). HBHG combined with ACEI/ARB may reduce serum creatinine (MD: −26.63 μmol/L, 95% CI: −47.91 to −5.35; low certainty), while TG combined with ACEI/ARB may reduce blood urea nitrogen (MD: −2.53 mmol/L, 95% CI: −4.54 to −0.52; moderate certainty). BL combined with ACEI/ARB may also improve hemoglobin (MD: 9.41 g/L, 95% CI: 8.18 to 10.64; low certainty). No convincing differences were observed in adverse drug reactions between most commercial Chinese polyherbal preparations combined with ACEI/ARB (very low to moderate certainty), although HK combined with ACEI/ARB showed a relatively higher incidence. Sensitivity analyses supported the robustness of findings. Egger’s test suggested potential publication bias for overall response rate and 24‐h urine protein quantification.

**Conclusion:**

In patients with IgAN, BL combined with ACEI/ARB may outperform other Chinese- Biomedicine regimens in reducing 24‐h urine protein quantification, with all combinations potentially superior to ACEI/ARB alone. However, evidence certainty remains low, highlighting the need for more rigorous, long-term RCTs for validation.

**Systematic Review Registration:**

https://www.crd.york.ac.uk/PROSPERO/view/CRD420251175045, PROSPERO: CRD420251175045.

## 1 Introduction

Immunoglobulin A nephropathy (IgAN), the most common primary glomerular disease globally (Jarrick et al., 2019; Schena and Nistor, 2018), progresses to end-stage renal disease (ESRD) in approximately 30% of patients. Treatment options remain limited and often yield unsatisfactory outcomes (Coppo, 2017; Wang et al., 2025). The cornerstone of conventional IgAN management involves renin-angiotensin system (RAS) inhibitors, namely, angiotensin-converting enzyme inhibitors (ACEIs) or angiotensin II receptor blockers (ARBs) (Kidney Disease: Improving Global Outcomes CKD Work Group, 2024; Cheng et al., 2009). Additionally, traditional immunosuppressive therapies, including glucocorticoids, cyclophosphamide, and tacrolimus, are available (Chen et al., 2022). However, their long-term adverse reactions and the risk of disease relapse after withdrawal have become new clinical challenges (Li et al., 2023; Zhang et al., 2023). Commercial Chinese polyherbal preparations (CCPPs) can significantly compensate for the limitations of Western therapy in IgAN. Despite this potential, clinicians lack evidence for selecting a first-choice agent. Some systematic reviews have summarized the benefits of CCPPs combined with ACEI/ARB. Nevertheless, these pairwise meta-analyses are typically constrained by small sample sizes and, more importantly, have failed to apply the Grading of Recommendations Assessment, Development and Evaluation (GRADE) framework to evaluate the certainty of the supporting evidence (Chen et al., 2022; Li et al., 2025).

To our knowledge, no network meta-analysis (NMA) has evaluated the efficacy and safety of six commonly used CCPPs (Bailing Capsule (BL), Huangkui Capsule (HK), Tripterygium Glycosides (TG), Jinshuibao Capsule (JSB), Huobahuagen Tablet (HBHG), and Shenyan Kangfu Tablet (SYKF)) in combination with ACEI/ARB therapy for IgAN. The optimal integrative treatment strategy combining Chinese and Biomedicine for IgA nephropathy remains undetermined. Therefore, we conducted a systematic review and NMA of relevant randomized controlled trials (RCTs) to evaluate the efficacy and safety of integrated CCPPs and Biomedicine (CCPPs-B) for IgAN, aiming to provide objective evidence-based findings for clinical practice.

## 2 Methods

This systematic review was registered in PROSPERO (CRD420251175045), and it was reported in accordance with the guidelines of the Preferred Reporting Items for Systematic Reviews and Meta-analyses (PRISMA) (Hutton et al., 2015). To improve transparency in the reporting of plant-derived medicinal interventions, we assessed the reporting completeness of the included commercial Chinese medicinal products using the ConPhyMP framework.

### 2.1 Search strategy and selection criteria

We searched PubMed, Embase, the Cochrane Library, the ClinicalTrials.gov, CNKI, Wanfang, VIP and SinoMed from database inception up to 20 October 2025, and screened the reference lists of relevant systematic reviews for additional trials. The search was conducted using a combination of controlled vocabulary (e.g., MeSH) and free-text terms. The search terms included “iga nephropath”, “tripterygium glycosides”, “bailing”, “jinshuibao”, “huangkui”, “huobahuagen”, “shenyankangfu”, and “randomized controlled trials” (Supplementary Appendix 1).

RCTs were included in this NMA if they met the following eligibility criteria: 1) Patients with IgA nephropathy confirmed by clearly defined diagnostic criteria; 2) The intervention group received treatment with CCPPs, including BL, JSB, HK, TG, HBHG, and SYKF combined with ACEI/ARB, while the control group received ACEI/ARB treatment alone; 3) Outcome measures included overall response rate (refers to the effective rate plus the marked effective rate, where “effective” means a 50%–70% reduction in 24-h urinary protein, and “marked effective” means 24-h urinary protein less than 0.3 g/day), 24-h urinary protein quantity (24hUPQ), serum creatinine (Scr), blood urea nitrogen (BUN), hemoglobin (Hb), and adverse drug reactions (ADRs). We excluded animal studies, studies without clearly defined diagnostic criteria, and studies for which full text was unavailable.

### 2.2 Study selection and data extraction

Using Endnote X9, pairs of reviewers (LZ, XTG, XLS, CMY and LZ*) independently screened titles and abstracts of all references and corresponding full-text documents initially deemed relevant. The identification of a retraction in any of the eligible publications led to the study’s exclusion from our analysis (Brown et al., 2022). Two reviewers (CMY and WNZ) independently extracted data on study characteristics, patient characteristics, intervention measures, and outcomes of interest. The process for resolving discrepancies involved discussion between the initial reviewers, with an additional reviewer (LZ*) being brought in to make a final decision if required.

### 2.3 Risk of bias assessment

Pairs of reviewers (LZ, XTG, XLS, CMY and LZ*) independently evaluated the risk of bias of included RCTs by using the Cochrane Risk of Bias Tool 2.0 (RoB 2.0) (Sterne et al., 2019) based on the following domains: bias arising from the randomisation process, bias due to deviations from intended interventions, bias due to missing outcome data, bias in measurement of the outcome, bias in selection of the reported result and other risk of bias. If the risk of bias assessment in all domains is judged as “low risk,” then the overall risk of bias is considered “low risk”. Conversely, if some domains are assessed as having “some concerns” and no domain is rated as “high risk,” the overall risk of bias is classified as “some concerns.” As long as one domain is evaluated as “high risk,” the overall risk of bias is deemed “high risk.”

### 2.4 Data analysis

Pairwise meta-analyses for all direct comparisons of each outcome were conducted utilizing the Hartung-Knapp-Sidik-Jonkman (HKSJ) random-effects model, implemented in R software (version 4.2.1). Using network packages in R (version 4.5.2), we performed random-effects NMA. For each outcome, the design-by-treatment model (global test) was employed to evaluate the coherence assumption across the entire network (Higgins et al., 2012). Indirect estimates were derived through node-splitting and back-calculation methods (König et al., 2013). To assess the inconsistency, we computed corresponding P values and deviance information criterion (DIC) for inconsistency testing (Dias et al., 2013).

For dichotomous outcomes, we calculated odds ratio (ORs) with 95% CIs for overall response rate and ADRs. For continuous outcomes, we calculated mean differences (MDs) with 95% CIs. In cases of missing SDs, these were imputed following the guidelines of the Cochrane Handbook (Higgins et al., 2022). Between-study heterogeneity was assessed using the I^2^ statistic and through visual inspection of forest plots. For meta-analyses comprising ten or more studies, we performed the Egger’s test for the outcomes by using the statistical software R (version 4.5.2) to assess publication bias, and also used the test to identify outliers (Egger et al., 1997). To assess the robustness of the pooled results and examine the sources of heterogeneity between studies, we conducted a sensitivity analysis. The relative performance of the treatments was inferred from the Surface Under the Cumulative Ranking Curve (SUCRA). SUCRA values range from 0% to 100%, where higher values represent a more favorable ranking.

We used the GRADE approach to assess certainty of evidence for the critical outcomes (Puhan et al., 2014; Brignardello-Petersen et al., 2018). By considering the risk of bias, inconsistency, indirectness, imprecision, publication bias, intransitivity, and incoherence, we graded the certainty of evidence into four level (Brignardello-Petersen et al., 2019; Guyatt et al., 2008): 1) very low, the estimate is subject to extreme uncertainty; 2) low, it is highly probable that further research will exert a considerable influence on our confidence in this assessment and is expected to revise it; 3) moderate, it is probable that further research will have an important impact on our confidence and might lead to a revised estimate; 4) high, it is improbable that further research will alter our confidence in the current estimate.

### 2.5 Description of investigated preparations

The interventions included in this review were CCPPs used in combination with ACEI/ARB-based biomedicine, including BL, HK, TG, JSB, HBHG, and SYKF. To improve reproducibility and transparency, we extracted all formulation-related information reported in the original trials, including dosage form, source of composition information, medicinal ingredients, single-herb or polyherbal, consistency of composition across included studies. We summarize this information in Supplementary Appendix 2.

The interventions included in this review were marketed commercial Chinese medicinal products with fixed formulations, rather than individualized herbal prescriptions or study-specific mixtures. Under routine manufacturing and regulatory practice, a given product is intended to have a stable composition under the same product name. Therefore, interventions were grouped according to the reported commercial product name and treated as the same marketed preparations across studies. In this respect, these products are conceptually closer to standardized marketed drug products than to flexible investigator-defined herbal formulations.

However, because this review was based on published randomized controlled trials, the level of formulation detail available for extraction depended on the reporting quality of the original articles. Although the products themselves are fixed commercial preparations, details such as manufacturer, batch number, and quality-control documentation were not consistently reported across all included studies.

### 2.6 Role of the funding source

The funder played no role in the design, conduct, analysis, or reporting of this study. All aspects of the research were performed independently by the authors.

## 3 Results

### 3.1 Search results and study characteristics

**FIGURE 1:**
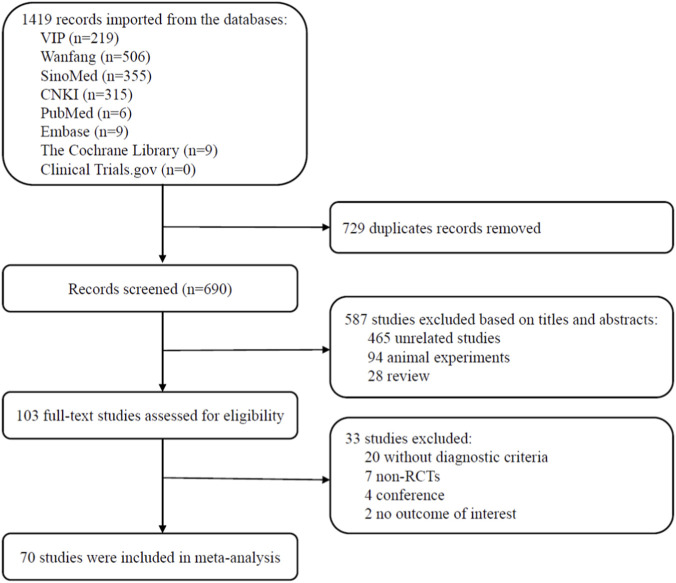
Study selection.

A total of 1419 records were identified through the electronic search. After 690 titles and abstracts underwent screening, 103 articles underwent full-text review. This process culminated in the final inclusion of 70 articles, which represented 70 unique RCTs (Zeng, 2021; Liu et al., 2018; Luo et al., 2023; Li, 2017; Yu and Zhu, 2015; Huang and He, 2011; Huo and Wu, 2016; Zhou, 2002; Zhang and Yang, 2016; Guan and Wu, 2005; Guo and Du, 2023; Wang and Lai, 2005; Zhao, 2013; Ma and Zhao, 2016; Li and Piao, 2017; Chen and Shou, 2015; Chen, 2019; Li et al., 2012; Han and Qiu, 2010; Su and Zhang, 2014; Tang, 2018; Zhang, 2021; Tang et al., 2009; Xu et al., 2009; Liang et al., 2017; Zhang and Xia, 2011; Zhang and Xia, 2010; Zhang et al., 2014; Lu, 2021; Zhang, 2014; Guo et al., 2019; Lu, 2020; Yang, 2010; Peng et al., 2010; Zhou et al., 2012; Yin, 2023; Yang and Chen, 2010; Lin et al., 2014; Sun, 2015; Zhang et al., 2010; Yang et al., 2016; Yang et al., 2013; Shi et al., 2015; Zhang et al., 2013; He et al., 2016; Huang et al., 2012; Wei et al., 2019; Yu and Huang, 2012; Fan et al., 2013; Lu et al., 2016; Yu, 2016; Yang et al., 2014; Liang et al., 2019; Hu, 2021; Fang et al., 2017; Li and Huang, 2021; Feng, 2020; Cai, 2018; Guan et al., 2015; Wang and Liu, 2018; Wei, 2019; Wang, 2020; Chen et al., 2021; Shen et al., 2009; Guo, 2017; Xiang et al., 2014; Cao and Chen, 2012; Wang, 2016; Xu, 2020; Shi et al., 2005) (Figure 1).

The basic information for each included RCTs was performed in Table 1. A total of 4855 patients were included, with a median age ranged from 21 to 70 years, the median duration time varied between 1 and 12 months, and almost of included RCTs reported the disease course of the patients spanning from 1 to 50 months.

**TABLE 1:** Basic characteristics of included studies in the systematic review.

Comparation	Study	No. (I/C)	Age (year)		Disease course (month)		Treatment duration (month)
			I	C	I	C	
BL + ACEI/ARB *vs.* ACEI/ARB	Zengcong 2021 (Zeng, 2021)	25/25	40.23 ± 4.18	39.75 ± 4.22	NR	NR	2
	Liulin 2018 (Liu et al., 2018)	30/30	35.78 ± 4.75	35.75 ± 4.72	3.32 ± 1.45	3.32 ± 1.46	NR
	Luoli 2023 (Luo et al., 2023)	40/40	47.84 ± 6.35	48.95 ± 7.26	3.14 ± 0.52	3.08 ± 0.45	3
	Liping 2017 (Li, 2017)	43/43	40.58 ± 6.39		15.46 ± 3.48		2
	Yuxuelei 2015 (Yu and Zhu, 2015)	50/50	37.9 ± 3.9	38.5 ± 4.2	9.9 ± 1.5	10.3 ± 1.6	2
	Huangqin 2011 (Huang and He, 2011)	33/17	NR		NR		3
	Huoyan 2016 (Huo and Wu, 2016)	31/35	36.5 ± 6.5		NR		4
	Zhouhongxing 2002 (Zhou, 2002)	18/14	NR		NR		3
	Zhangnan 2016 (Zhang and Yang, 2016)	48/48	36.59 ± 15.22	35.31 ± 13.57	1.80 ± 1.40	1.87 ± 1.35	1
HBHG + ACEI/ARB *vs.* ACEI/ARB	Guanxiaodong 2005 (Guan and Wu, 2005)	32/30	31.2 ± 6.8	30.4 ± 6.2	13.2 ± 9.1	12.8 ± 8.7	3
	Guomin 2023 (Guo and Du, 2023)	50/50	42.06 ± 3.84	41.16 ± 3.33	72.12 ± 1.52	68.28 ± 1.45	6
	Wangkunming 2005 (Wang and Lai, 2005)	54/18	29.8 ± 8.5		2.8 ± 1.4		2
HK + ACEI/ARB *vs.* ACEI/ARB	Zhaojian 2013 (Zhao, 2013)	41/41	34 ± 9	33 ± 8	7 ± 3	8 ± 3	2
	Mafei 2016 (Ma and Zhao, 2016)	40/40	36.2 ± 12.4		13.6 ± 2.3		3
	Limingyu 2017 (Li and Piao, 2017)	35/35	35.0 ± 11.1	32.0 ± 12.1	14.2 ± 3.6	14.3 ± 3.7	3
	Chenjianjun 2015 (Chen and Shou, 2015)	30/30	39.2 ± 11.2	21.21 ± 10.64	38.8 ± 10.6	21.47 ± 10.56	2
	Chenxuelian 2019 (Chen, 2019)	40/40	28.58 ± 6.36	27.84 ± 5.36	10.69 ± 2.16	9.56 ± 2.98	2
	Liliusheng 2012 (Li et al., 2012)	30/30	32.8 ± 12.7	14.4 ± 3.8	34.5 ± 11.7	14.5 ± 3.7	3
	Hanyarong 2010 (Han and Qiu, 2010)	29/26	37 ± 17.4	38 ± 16.3	NR		3
	Supofeng 2014 (Su and Zhang, 2014)	24/24	34.4 ± 7.9	34.2 ± 8.1	13.4 ± 5.4	13.1 ± 7.6	3
	Tangyi 2018 (Tang, 2018)	34/34	37.5 ± 3.6	37.2 ± 3.2	11.8 ± 1.6	11.2 ± 1.2	3
	Zhangchunzhao 2021 (Zhang, 2021)	30/30	46.22 ± 4.26	46.23 ± 4.36	NR		3
	Tangweigang 2009 (Tang et al., 2009)	70/50	30.18 ± 16.26	31.25 ± 15.36	3.26 ± 2.85	3.58 ± 2.68	2
​	Xuke 2009 (Xu et al., 2009)	42/38	36.4 ± 10.8	35.8 ± 12.4	NR		4
	Liangyan 2017 (Liang et al., 2017)	26/26	37.1 ± 11.7	38.7 ± 12.1	NR		6
	Zhangning 2011 (Zhang and Xia, 2011)	32/30	38 ± 10	37 ± 11	NR		3
	Zhangning 2010 (Zhang and Xia, 2010)	52/50	44 ± 16.7	42 ± 17.8	NR		3
	Zhangchen 2014 (Zhang et al., 2014)	30/30	NR		NR		2
	Luhuiqin 2021 (Lu, 2021)	78/78	49.67 ± 6.16	51.06 ± 5.34	10.28 ± 2.86	11.26 ± 3.02	6
	Zhangwei 2014 (Zhang, 2014)	36/36	35.9 ± 10.2		19.2 ± 0.9		2
	Guoyuqiang 2019 (Guo et al., 2019)	30/30	44.5 ± 6.1	45.2 ± 6.0	5.1 ± 3.3	5.3 ± 3.0	3
	Luwenjuan 2020 (Lu, 2020)	25/25	58.2 ± 9.1	57.5 ± 8.9	NR		3
	Yangjuhong 2010 (Yang, 2010)	31/30	NR	NR	NR		6
	Pengtao 2010 (Peng et al., 2010)	28/25	39.9 ± 7.9	41.7 ± 8.7	NR		3
	Zhouhuilan 2012 (Zhou et al., 2012)	30/30	NR		NR		2
	Yinyong 2023 (Yin, 2023)	45/45	35.68 ± 7.12	36.09 ± 6.88	36.54 ± 8.33	37.11 ± 7.95	3
	Yangliu 2010 (Yang and Chen, 2010)	21/21	26.8 ± 8.3		NR		3
JSB + ACEI/ARB *vs.* ACEI/ARB	Linlijuan 2014 (Lin et al., 2014)	30/30	35.2 ± 10.8		20.4 ± 10.2	21.0 ± 10.3	2
	Sunxuehui 2015 (Sun, 2015)	53/50	32 ± 7	33 ± 6	NR		6
	Zhangyongxiu 2010 (Zhang et al., 2010)	53/49	35.9 ± 8.7	37.1 ± 10.2	NR		3
SYKF + ACEI/ARB *vs.* ACEI/ARB	Yangzhongmin 2016 (Yang et al., 2016)	60/60	42.8 ± 10.6	43.7 ± 11.2	NR		4
	Yangpo 2013 (Yang et al., 2013)	22/21	31.6 ± 10.4		64.8 ± 1.3		3
	Shifeng 2015 (Shi et al., 2015)	20/20	22.3	​	NR		2
	Zhangxinzhi 2013 (Zhang et al., 2013)	32/30	38.6 ± 4.28		4.5 ± 20.5		2
	Helijuan 2016 (He et al., 2016)	20/20	26.7 ± 3.4	33.4 ± 6.2	5.2 ± 3.1	4.2 ± 3.1	2
	Huangjing 2012 (Huang et al., 2012)	25/25	NR		NR		3
TG + ACEI/ARB *vs.* ACEI/ARB	Weilan 2019 (Wei et al., 2019)	51/47	31.2 ± 4.3	32.4 ± 5.0	20.4 ± 0.2	18 ± 0.3	3
	Yuxufeng 2012 (Yu and Huang, 2012)	20/22	36.9 ± 9.8	37.3 ± 10.5	NR		3
	Fanye 2013 (Fan et al., 2013)	20/20	52.3 ± 12.1	​	NR		NR
	Luxiaomei 2016 (Lu et al., 2016)	55/54	41.36 ± 11.76	37.89 ± 11.14	NR		12
	Yutao 2016 (Yu, 2016)	30/30	46.1 ± 9.4	45.2 ± 5.7	NR		3
	Yangzhongmin 2014 (Yang et al., 2014)	32/31	50.01 ± 10.12	49.3 ± 10.6	NR		3
	Liangyan 2019 (Liang et al., 2019)	46/40	45.5 ± 12.7	46.3 ± 11.4	38.7 ± 16.8	35.8 ± 15.3	3
	Hujiane 2021 (Hu, 2021)	48/48	49.40 ± 9.72	48.74 ± 9.56	25.41 ± 8.12	25.61 ± 8.13	2
	Fanghui 2017 (Fang et al., 2017)	40/40	66.2 ± 4.1	65.2 ± 4.0	11.90 ± 0.22	11.80 ± 0.32	12
	Liyanfeng 2021 (Li and Huang, 2021)	55/55	41.47 ± 12.51	41.52 ± 12.33	27.6 ± 0.95	26.2 ± 0.97	6
	Fengwei 2020 (Feng, 2020)	45/45	53.53 ± 9.52		NR		3
	Caiyuping 2018 (Cai, 2018)	34/34	46.12 ± 9.05	45.78 ± 8.83	NR		6
	Guanyibiao 2015 (Guan et al., 2015)	34/31	37.25 ± 6.73	36.72 ± 5.92	23.02 ± 3.56	22.18 ± 2.73	3
	Wangdan 2018 (Wang and Liu, 2018)	33/33	37.24 ± 6.72	37.71 ± 5.91	NR		NR
	Weijiwei 2019 (Wei, 2019)	35/35	39.57 ± 5.16	37.65 ± 5.58	1.35 ± 1.28	1.28 ± 1.23	3
	Wangyanhua 2020 (Wang, 2020)	17/17	42.61 ± 4.22	43.75 ± 3.92	NR		6
	Chendu 2021 (Chen et al., 2021)	40/40	37.12 ± 2.13	36.99 ± 2.14	18.11 ± 1.52	18.26 ± 1.55	6
	Shenshuijuan 2009 (Shen et al., 2009)	26/26	32.48 ± 10.12		NR		6
	Guobaoqin 2017 (Guo, 2017)	15/15	54.1 ± 8.9	53.7 ± 8.5	NR		3
	Xiangqiong 2014 (Xiang et al., 2014)	30/30	50.3 ± 9.6	51.3 ± 8.2	2.7 ± 1.9	2.6 ± 1.7	3
	Caoshan 2012 (Cao and Chen, 2012)	12/11	NR		NR		3
	Wangxiaoyan 2016 (Wang, 2016)	41/41	33.52 ± 5.30	35.21 ± 4.46	NR		3
	Xulei 2020 (Xu, 2020)	31/31	49.07 ± 8.04	48.57 ± 7.88	NR		2
​	Shiyongjun 2005 (Shi et al., 2005)	19/13	37.5		NR		12

NR, not application; ACEI, angiotensin-converting enzyme inhibitor; ARB, angiotensin receptor blocker; BL, bailing capsule; HBHG, huobahuagen tablet; HK, huangkui capsule; TG, tripterygium glycosides; JSB, jinshuibao capsule; SYKF, shenyan kangfu tablet.

### 3.2 Risk of bias assessment

The risk of bias for the included RCTs was provided in Supplementary Appendix 3. Sixteen studies (Han and Qiu, 2010; Su and Zhang, 2014; Liang et al., 2017; Zhang and Xia, 2011; Zhang, 2014; Guo et al., 2019; Yang, 2010; Zhou et al., 2012; Sun, 2015; Zhang et al., 2013; Huang et al., 2012; Yu and Huang, 2012; Liang et al., 2019; Shen et al., 2009; Cao and Chen, 2012; Shi et al., 2005) were classified as high risk of bias due to inadequate allocation concealment and lack of blinding. Nineteen studies (Zeng, 2021; Liu et al., 2018; Luo et al., 2023; Huang and He, 2011; Huo and Wu, 2016; Zhou, 2002; Zhang and Yang, 2016; Guan and Wu, 2005; Ma and Zhao, 2016; Li and Piao, 2017; Tang, 2018; Xu et al., 2009; Zhang and Xia, 2010; Zhang et al., 2014; Peng et al., 2010; Zhang et al., 2010; Yang et al., 2013; Shi et al., 2015; He et al., 2016) were rated as probably high risk of bias primarily due to deviations from intended intervention. Thirty-three studies (Li, 2017; Yu and Zhu, 2015; Guo and Du, 2023; Wang and Lai, 2005; Zhao, 2013; Chen and Shou, 2015; Chen, 2019; Li et al., 2012; Zhang, 2021; Tang et al., 2009; Lu, 2021; Lu, 2020; Yin, 2023; Yang and Chen, 2010; Lin et al., 2014; Yang et al., 2016; Wei et al., 2019; Fan et al., 2013; Yu, 2016; Yang et al., 2014; Hu, 2021; Fang et al., 2017; Feng, 2020; Cai, 2018; Guan et al., 2015; Wang and Liu, 2018; Wei, 2019; Wang, 2020; Chen et al., 2021; Guo, 2017; Xiang et al., 2014; Wang, 2016; Xu, 2020) were categorized as probably low risk of bias, while only two studies (Lu et al., 2016; Li and Huang, 2021) were rated as low risk. Overall, the quality of the included studies was not high. However, in terms of “Bias due to missing outcome data”, only two studies (Huang and He, 2011; Zhou, 2002) were assessed as probably high risk of bias, indicating virtually no data integrity issues identified in the included research.

### 3.3 Network meta-analysis (NMA)

**FIGURE 2:**
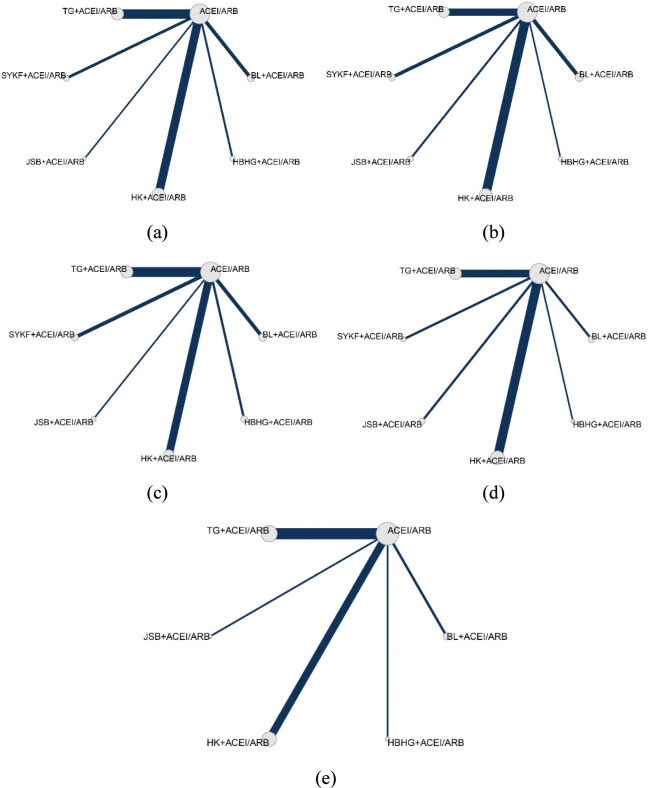
Network plot for overall response rate **(a)**, 24hUPQ **(b)**, blood urea nitrogen **(c)**, hemoglobin **(d)**, adverse drug reactions **(e)** (Notes: Circle size corresponds to participant count; connecting lines and their width indicate direct comparisons and study volume, respectively).

Seventy studies were included in the NMA (Zeng, 2021; Liu et al., 2018; Luo et al., 2023; Li, 2017; Yu and Zhu, 2015; Huang and He, 2011; Huo and Wu, 2016; Zhou, 2002; Zhang and Yang, 2016; Guan and Wu, 2005; Guo and Du, 2023; Wang and Lai, 2005; Zhao, 2013; Ma and Zhao, 2016; Li and Piao, 2017; Chen and Shou, 2015; Chen, 2019; Li et al., 2012; Han and Qiu, 2010; Su and Zhang, 2014; Tang, 2018; Zhang, 2021; Tang et al., 2009; Xu et al., 2009; Liang et al., 2017; Zhang and Xia, 2011; Zhang and Xia, 2010; Zhang et al., 2014; Lu, 2021; Zhang, 2014; Guo et al., 2019; Lu, 2020; Yang, 2010; Peng et al., 2010; Zhou et al., 2012; Yin, 2023; Yang and Chen, 2010; Lin et al., 2014; Sun, 2015; Zhang et al., 2010; Yang et al., 2016; Yang et al., 2013; Shi et al., 2015; Zhang et al., 2013; He et al., 2016; Huang et al., 2012; Wei et al., 2019; Yu and Huang, 2012; ; Fan et al., 2013; Lu et al., 2016; Yu, 2016; Yang et al., 2014; Liang et al., 2019; Hu, 2021; Fang et al., 2017; Li and Huang, 2021; Feng, 2020; Cai, 2018; Guan et al., 2015; Wang and Liu, 2018; Wei, 2019; Wang, 2020; Chen et al., 2021; Shen et al., 2009; Guo, 2017; Xiang et al., 2014; Cao and Chen, 2012; Wang, 2016; Xu, 2020; Shi et al., 2005). Network plots for the major outcomes are shown in Figure 2. Between-study heterogeneity varied across outcomes and comparisons and was mainly observed in some continuous outcomes (Supplementary Appendix 4), whereas no significant inconsistency was detected for any outcome (Supplementary Appendix 5). The GRADE summary of findings was performed in Supplementary Appendix 6. We judged the certainty of evidence to be low or very low for most of outcomes.

The NMA of overall response rate included 51 studies (Zeng, 2021; Liu et al., 2018; Luo et al., 2023; Yu and Zhu, 2015; Huang and He, 2011; Zhou, 2002; Guo and Du, 2023; Wang and Lai, 2005; Zhao, 2013; Ma and Zhao, 2016; Li and Piao, 2017; Chen, 2019; Li et al., 2012; Han and Qiu, 2010; Su and Zhang, 2014; Tang, 2018; Zhang, 2021; Tang et al., 2009; Xu et al., 2009; Zhang et al., 2014; Lu, 2021; Zhang, 2014; Guo et al., 2019; Lu, 2020; Yang, 2010; Zhang et al., 2014; Lu, 2021; Zhang, 2014; Guo et al., 2019; Lu, 2020; Yang, 2010; Zhang et al., 2014; Lu, 2021; Zhang, 2014; Guo et al., 2019; Lu, 2020; Yang, 2010; Zhang et al., 2014; Lu, 2021; Zhang, 2014; Guo et al., 2019; Lu, 2020; Yang, 2010; Shi et al., 2015; Zhang et al., 2013; He et al., 2016; Wei et al., 2019; Yu and Huang, 2012; Fan et al., 2013; Lu et al., 2016; Yu, 2016; Yang et al., 2014; Liang et al., 2019; Wei et al., 2019; Yu and Huang, 2012; Fan et al., 2013; Lu et al., 2016; Yu, 2016; Yang et al., 2014; Liang et al., 2019; Cai, 2018; Guan et al., 2015; Wang and Liu, 2018; Wei, 2019; Wang, 2020; Chen et al., 2021; Shen et al., 2009; Guo, 2017; Xiang et al., 2014; Cao and Chen, 2012; Wang, 2016; Cai, 2018; Guan et al., 2015; Wang and Liu, 2018; Wei, 2019; Wang, 2020; Chen et al., 2021; Shen et al., 2009; Guo, 2017; Xiang et al., 2014; Cao and Chen, 2012; Wang, 2016) of BL combined with AECI/ARB, HBHG combined with ACEI/ARB, HK combined with ACEI/ARB, JSB combined with ACEI/ARB, SYKF combined with ACEI/ARB or TG combined with ACEI/ARB, involving 3518 patients. The results showed that compared with ACEI/ARB alone, CCPPs combined with ACEI/ARB significantly improved the overall response rate, with the effect of BL combined with ACEI/ARB being the most significant (OR: 3.27, 95% CI: 2.21–4.84), and the certainty of evidence was moderate (detailed results can be seen in Supplementary Appendix 6.1). When compared with each other, the OR ranged from 0.72 to 1.26, and the certainty of evidence was very low (see Supplementary Appendix 6.1). According to their respective SUCRA values, it can be observed that regardless of whether compared with ACEI/ARB alone or with each other, the BL combined with ACEI/ARB regimen demonstrated the most significant therapeutic effect (see Supplementary Appendix 7.1, Supplementary Appendix 8).

The NMA of 24hUPQ included 52 studies (Liu et al., 2018; Luo et al., 2023; Li, 2017; Yu and Zhu, 2015; Huang and He, 2011; Huo and Wu, 2016; Liu et al., 2018; Luo et al., 2023; Li, 2017; Yu and Zhu, 2015; Huang and He, 2011; Huo and Wu, 2016; Liu et al., 2018; Luo et al., 2023; Li, 2017; Yu and Zhu, 2015; Huang and He, 2011; Huo and Wu, 2016; Liu et al., 2018; Luo et al., 2023; Li, 2017; Yu and Zhu, 2015; Huang and He, 2011; Huo and Wu, 2016; Liu et al., 2018; Luo et al., 2023; Li, 2017; Yu and Zhu, 2015; Huang and He, 2011; Huo and Wu, 2016; Liu et al., 2018; Luo et al., 2023; Li, 2017; Yu and Zhu, 2015; Huang and He, 2011; Huo and Wu, 2016; Han and Qiu, 2010; Su and Zhang, 2014; Tang, 2018; Zhang, 2021; Tang et al., 2009; Xu et al., 2009; Liang et al., 2017; Zhang and Xia, 2011; Zhang and Xia, 2010; Zhang et al., 2014; Lu, 2021; Zhang, 2014; Lu, 2020; Yang, 2010; Peng et al., 2010; Yin, 2023; Yang and Chen, 2010; Lin et al., 2014; Sun, 2015; Zhang et al., 2010; Yang et al., 2016; Yang et al., 2013; Shi et al., 2015; Zhang et al., 2013; He et al., 2016; Huang et al., 2012; Yin, 2023; Yang and Chen, 2010; Lin et al., 2014; Sun, 2015; Zhang et al., 2010; Yang et al., 2016; Yang et al., 2013; Shi et al., 2015; Zhang et al., 2013; He et al., 2016; Huang et al., 2012; Yu, 2016; Yang et al., 2014; Liang et al., 2019; Hu, 2021; Fang et al., 2017; Feng, 2020; Cai, 2018; Guan et al., 2015; Wang, 2020; Chen et al., 2021; Shen et al., 2009; Wang, 2020; Chen et al., 2021; Shen et al., 2009; Wang, 2020; Chen et al., 2021; Shen et al., 2009; Wang, 2020; Chen et al., 2021; Shen et al., 2009) of BL combined with AECI/ARB, HBHG combined with ACEI/ARB, HK combined with ACEI/ARB, JSB combined with ACEI/ARB, SYKF combined with ACEI/ARB or TG combined with ACEI/ARB, involving 3629 patients. The results showed that compared with ACEI/ARB alone, CCPPs combined with ACEI/ARB significantly reduced the 24hUPQ, with the effect of BL combined with ACEI/ARB being the most significant (MD: −0.83 g, 95% CI: −1.21 to −0.44), and the certainty of evidence was moderate (detailed results can be seen in Supplementary Appendix 6.2). When compared with each other, the MD ranged from −0.17 g to 0.35 g, and the certainty of evidence was very low (see Supplementary Appendix 6.2). BL combined with ACEI/ARB regimen demonstrated the most significant therapeutic effect based on their respective SUCRA values (see Supplementary Appendix 7.2, Supplementary Appendix 8).

The NMA of Scr included 40 studies (Liu et al., 2018; Luo et al., 2023; Li, 2017; Yu and Zhu, 2015; Huang and He, 2011; Guan and Wu, 2005; Guo and Du, 2023; Wang and Lai, 2005; Zhao, 2013; Li et al., 2012; Han and Qiu, 2010; Zhang, 2021; Tang et al., 2009; Liang et al., 2017; Zhang et al., 2014; Lu, 2021; Zhang, 2014; Yang, 2010; Yang and Chen, 2010; Lin et al., 2014; Sun, 2015; Yang et al., 2016; Yang et al., 2013; Zhang et al., 2013; He et al., 2016; Huang et al., 2012; Yu and Huang, 2012; Liang et al., 2019; Hu, 2021; Fang et al., 2017; Li and Huang, 2021; Feng, 2020; Cai, 2018; Guan et al., 2015; Wei, 2019; Wang, 2020; Chen et al., 2021; Shen et al., 2009; Xiang et al., 2014; Shi et al., 2005) of BL combined with AECI/ARB, HBHG combined with ACEI/ARB, HK combined with ACEI/ARB, JSB combined with ACEI/ARB, SYKF combined with ACEI/ARB or TG combined with ACEI/ARB, involving 2867 patients. The results showed that compared with ACEI/ARB alone, CCPPs combined with ACEI/ARB significantly reduced the Scr, with the effect of HBHG combined with ACEI/ARB being the most significant (MD: −26.63 μmol/L, 95%CI: −47.91 to −5.35), and the certainty of evidence was low (detailed results can be seen in Supplementary Appendix 6.3). When compared with each other, the MD ranged from −12.38 μmol/L to 18.60 μmol/L, and the certainty of evidence was very low (see Supplementary Appendix 6.3). However, it is worth noting that BL combined with ACEI/ARB regimen demonstrated the most significant therapeutic effect based on their respective SUCRA values (see Supplementary Appendix 7.3, Supplementary Appendix 8).

The NMA of BUN included 20 studies (Liu et al., 2018; Luo et al., 2023; Huang and He, 2011; Guo and Du, 2023; Wang and Lai, 2005; Zhao, 2013; Li et al., 2012; Han and Qiu, 2010; Zhang et al., 2014; Lu, 2021; Zhang, 2014; Yang et al., 2016; Huang et al., 2012; Liang et al., 2019; Hu, 2021; Fang et al., 2017; Li and Huang, 2021; Cai, 2018; Wang, 2020; Xiang et al., 2014) of BL combined with AECI/ARB, HBHG combined with ACEI/ARB, HK combined with ACEI/ARB, SYKF combined with ACEI/ARB or TG combined with ACEI/ARB, involving 1548 patients. The results showed that compared with ACEI/ARB alone, CCPPs combined with ACEI/ARB significantly reduced the BUN, with the effect of TG combined with ACEI/ARB being the most significant (MD: −2.53 mmol/L, 95% CI: −4.54 to −0.52), and the certainty of evidence was moderate (detailed results can be seen in Supplementary Appendix 6.4). When compared with each other, the MD ranged from −2.22 mmol/L to 1.46 mmol/L, and the certainty of evidence was very low (see Supplementary Appendix 6.4). Based on their respective SUCRA values, TG combined with ACEI/ARB regimen demonstrated the most significant therapeutic effect (see Supplementary Appendix 7.4, Supplementary Appendix 8).

The NMA of Hb included 29 studies (Li, 2017; Yu and Zhu, 2015; Guan and Wu, 2005; Li et al., 2012; Han and Qiu, 2010; Su and Zhang, 2014; Tang, 2018; Zhang, 2021; Tang et al., 2009; Liang et al., 2017; Zhang and Xia, 2010; Zhang et al., 2014; Zhang, 2014; Yang, 2010; Yin, 2023; Lin et al., 2014; Sun, 2015; Zhang et al., 2013; He et al., 2016; Yu and Huang, 2012; Liang et al., 2019; Fang et al., 2017; Cai, 2018; Guan et al., 2015; Wei, 2019; Chen et al., 2021; Shen et al., 2009; Xiang et al., 2014; Shi et al., 2005) of BL combined with AECI/ARB, HBHG combined with ACEI/ARB, HK combined with ACEI/ARB, SYKF combined with ACEI/ARB or TG combined with ACEI/ARB, involving 1994 patients. The results showed that compared with ACEI/ARB alone, CCPPs combined with ACEI/ARB significantly improved the Hb, with the effect of BL combined with ACEI/ARB being the most significant (MD: 9.41 g/L, 95% CI: 8.18–10.64), and the certainty of evidence was low (detailed results can be seen in Supplementary Appendix 6.5). When compared with each other, the MD ranged from −6.96 g/L to 4.73 g/L, and the certainty of evidence was very low (see Supplementary Appendix 6.5). Based on their respective SUCRA values, BL combined with ACEI/ARB regimen demonstrated the most significant therapeutic effect (see Supplementary Appendix 7.5, Supplementary Appendix 8).

The NMA of ADRs included 29 studies (Huang and He, 2011; Zhang and Yang, 2016; Guan and Wu, 2005; Ma and Zhao, 2016; Li and Piao, 2017; Chen and Shou, 2015; Chen, 2019; Han and Qiu, 2010; Zhang et al., 2014; Lu, 2021; Zhang, 2014; Zhou et al., 2012; Yin, 2023; Lin et al., 2014; Wei et al., 2019; Yu and Huang, 2012; Lu et al., 2016; Yang et al., 2014; Liang et al., 2019; Fang et al., 2017; Li and Huang, 2021; Guan et al., 2015; Wang and Liu, 2018; Wei, 2019; Shen et al., 2009; Xiang et al., 2014; Cao and Chen, 2012; Xu, 2020; Shi et al., 2005) of BL combined with AECI/ARB, HBHG combined with ACEI/ARB, HK combined with ACEI/ARB, JSB combined with ACEI/ARB or TG combined with ACEI/ARB, involving 2059 patients. There were no convincing differences in any ADRs among these drugs. The certainty of evidence was ranged from very low to moderate (see Supplementary Appendix 6.6). Based on their respective SUCRA values, it can be observed that the incidence of ADRs varies little among the different treatment measures, with the HK group showing a relatively higher incidence of ADRs (see Supplementary Appendix 7.6, 8). The most commonly reported ADRs across all groups were mild gastrointestinal discomfort (nausea, abdominal distension, diarrhea), transient dizziness, and mild elevation of liver transaminases. All reported ADRs were mild to moderate in severity and resolved spontaneously without discontinuation of treatment. No fatal or life-threatening serious adverse events were reported in any of the included trials (see Supplementary Appendix 9).

### 3.4 Sensitivity analysis and publication bias

A sensitivity analysis was conducted to evaluate the robustness of the NMA results. This was achieved by iteratively removing each of the 70 included studies (Zeng, 2021; Liu et al., 2018; Luo et al., 2023; Li, 2017; Yu and Zhu, 2015; Huang and He, 2011; Huo and Wu, 2016; Zhou, 2002; Zhang and Yang, 2016; Guan and Wu, 2005; Guo and Du, 2023; Wang and Lai, 2005; Zhao, 2013; Ma and Zhao, 2016; Li and Piao, 2017; Chen and Shou, 2015; Chen, 2019; Li et al., 2012; Han and Qiu, 2010; Su and Zhang, 2014; Tang, 2018; Zhang, 2021; Tang et al., 2009; Xu et al., 2009; Liang et al., 2017; Zhang and Xia, 2011; Zhang and Xia, 2010; Zhang et al., 2014; Lu, 2021; Zhang, 2014; Guo et al., 2019; Lu, 2020; Yang, 2010; Peng et al., 2010; Zhou et al., 2012; Yin, 2023; Yang and Chen, 2010; Lin et al., 2014; Sun, 2015; Zhang et al., 2010; Yang et al., 2016; Yang et al., 2013; Shi et al., 2015; Zhang et al., 2013; He et al., 2016; Huang et al., 2012; Wei et al., 2019; Yu and Huang, 2012; ; Fan et al., 2013; Lu et al., 2016; Yu, 2016; Yang et al., 2014; Liang et al., 2019; Hu, 2021; Fang et al., 2017; Li and Huang, 2021; Feng, 2020; Cai, 2018; Guan et al., 2015; Wang and Liu, 2018; Wei, 2019; Wang, 2020; Chen et al., 2021; Shen et al., 2009; Guo, 2017; Xiang et al., 2014; Cao and Chen, 2012; Wang, 2016; Xu, 2020; Shi et al., 2005). The stability of the overall results is evidenced by the fact that omitting any individual study did not significantly alter the drug’s therapeutic efficacy, further affirming the consistency of the findings (see Supplementary Appendix 10). Additionally, Egger’s test was performed to assess publication bias for the following outcomes: overall response rate, 24hUPQ, Scr, BUN, Hb, and ADRs. The results indicated significant publication bias for overall response rate and 24hUPQ (Egger’s test p < 0.05). For all other outcomes, the Egger’s test results were non-significant (p > 0.05) (see Table 2).

**TABLE 2:** The results of Egger’s test.

Outcomes	Overall response rate	24hUPQ	Scr	BUN	Hb	ADRs
P	0.001	0.01	0.06	0.14	0.89	0.23

24hUPQ, 24-h urinary protein quantification; Scr, serum creatinine; BUN, blood urea nitrogen; Hb, hemoglobin; ADRs, adverse drug reactions.

## 4 Discussion

This NMA systematically compared the efficacy and safety of six CCPPs combined with ACEI/ARB in 4,855 IgAN patients. The BL combined with ACEI/ARB regimen demonstrated the highest probability of being the best for improving the overall response rate, reducing 24hUPQ, and increasing Hb level, with SUCRA values consistently high. In contrast, the TG combined with ACEI/ARB regimen showed a unique advantage in reducing BUN level. It is noteworthy that none of the combination regimens showed clinically significant differences in safety profiles, although the quality of evidence was generally low or very low.

To some extent, our findings corroborate and extend the existing body of evidene on integrated CCPPs-B for IgAN. The study confirms this trend utilizing NMA and further quantifies the relative efficacy among different CCPPs, thereby providing more nuanced ranking information for clinical decision-making. However, similar to the findings of Gao et al. (2024) in the context of anti-influenza viral drugs, the core conclusions of our study are also plagued by very low quality of evidence. Although studies have shown that combination therapy of CCPPs-B may benefit kidney protection (Chen et al., 2022; Li et al., 2025), the included studies did not undergo long-term follow-up, resulting in limited understanding of the true effects of these combination regimens on hard endpoints such as delaying estimated glomerular filtration rate (eGFR) decline and reducing the incidence of end-stage renal disease.

Compared with previous reviews, a major strength of this study is that we evaluated multiple CCPPs combined with ACEI/ARB within a single network meta-analysis, rather than focusing on one specific intervention or relying only on pairwise comparisons. This allowed a more comprehensive comparison of the relative efficacy and safety of different treatment options. In addition, some previous pharmacological and experimental studies provide mechanistic support for our findings. TG have been reported to exert anti-inflammatory and immunomodulatory effects and to alleviate renal injury (Chen et al., 2022). HK capsule, derived from Abelmoschus manihot, may have anti-inflammatory, anti-oxidative, and anti-fibrotic properties and has shown renoprotective potential in kidney disease (Pei and Li, 2021; Yuan et al., 2023). BL capsule and JSB capsule, both related to Cordyceps sinensis, have also been reported to improve renal function and regulate immune and inflammatory responses (Wang et al., 2024; Fan et al., 2025). Given the important roles of inflammation, immune dysregulation, and fibrosis in IgA nephropathy, these findings provide a plausible biological basis for the observed clinical benefits. However, these mechanistic data are supportive rather than confirmatory, and the conclusions of this study remain primarily based on comparative clinical evidence.

This study had three key methodological strengths. First, the application of a NMA transcended the limitations of conventional pairwise comparisons. By constructing an evidence network containing six intervention measures, we quantified and ranked the relative efficacy of different integrated CCPPs-B regimens, thereby providing more refined evidence for clinical decision-making. Second, the study rigorously adhered to the PRISMA guidelines (Hutton et al., 2015), employing the Cochrane Risk of Bias tool (Sterne et al., 2019) to assess the included studies and the GRADE framework to evaluate the certainty of evidence (Puhan et al., 2014; Brignardello-Petersen et al., 2018). Finally, the combined presentation of SUCRA values with traditional effect sizes not only illustrates the comprehensive ranking probabilities of each regimen but also provides the magnitude of clinical benefit, thereby enhancing the practical applicability of the findings.

However, the limitations of this study should not be overlooked. First and foremost, the methodological quality of the included studies was generally low. Among the 70 included studies, only two were rated as having a low risk of bias. The vast majority exhibited a high or unclear risk of bias in key domains such as random sequence generation, allocation concealment, and blinding, which likely led to an overestimation of the treatment effect. Second, we observed pronounced statistical heterogeneity. For key continuous outcomes, such as 24hUPQ and Scr, high I^2^ values were consistently observed. This heterogeneity likely stems from intrinsic variations in the production processes, chemical composition, and dosage of the CCPPs themselves, as well as differences in the etiology, disease stages, and concomitant treatments among the enrolled patients. Although no significant statistical inconsistency was detected, the clinical and methodological heterogeneity nevertheless undermines the robustness of the pooled results. Third, Egger’s test suggested possible publication bias for two outcomes, both of which were important endpoints favoring BL. This raises the possibility that the observed benefit of BL may have been exaggerated by small-study effects or selective publication. Therefore, the superiority of BL should be interpreted with caution. Furthermore, the results of Egger’s test for the overall response rate suggest the possible presence of publication bias, indicating that positive results might have been more likely to be published. Finally, there were important limitations regarding exploration of heterogeneity. Although sensitivity analyses showed that the main findings were robust to the exclusion of individual studies, we did not perform formal subgroup analyses based on factors such as risk of bias, treatment duration, or sample size. This decision was made for methodological reasons. Specifically, the number of studies available for several treatment comparisons and outcomes was limited, and further stratification would have resulted in sparse subnetworks, unstable estimates, and reduced interpretability. In addition, key stratification variables were incompletely and inconsistently reported across the included trials, and simple study-level subgrouping would have been unlikely to adequately explain the observed heterogeneity. Therefore, we considered it more appropriate to address these issues through cautious interpretation, sensitivity analyses, and certainty rating using the GRADE framework. Nevertheless, the lack of formal subgroup analyses limits our ability to determine whether specific patient subgroups may benefit more from particular interventions.

In clinical practice, given the current low-certainty evidence, any therapeutic decision must be approached with caution. For IgAN patients considering integrated CCPPs-B therapy, the BL combined with ACEI/ARB regimen demonstrates potential in reducing proteinuria and improving Hb, yet the level of evidence remains low. Clinicians should recognize that the existing evidence falls far short of establishing any combination therapy as a standard treatment plan.

This NMA underscores the imperative for future large-scale, high-quality, long-term RCTs. Future investigations should prioritize: 1) enhance methodological rigor through strict implementation of randomization, allocation concealment, and blinding, particularly for outcome assessors; 2) focus on patient-important long-term hard endpoints, such as renal failure, cardiovascular events and all-cause mortality; 3) Standardize the intervention measures and clarify the standardized ingredients, dosage and course of treatment of CCPPs and simple preparations to reduce heterogeneity; 4) conduct pre-specified subgroup analyses to explore treatment effect variations across different clinical characteristics and traditional Chinese medicine syndrome types, thereby facilitating precision medicine.

## 5 Conclusion

Based on current evidence from randomized trials, in patients with IgAN, BL + ACEI/ARB appeared to show favorable effects in some key outcomes, however, given the potential publication bias detected for outcomes supporting BL, this apparent advantage should be interpreted cautiously and requires confirmation in future high-quality studies. While all combined regimens may offer advantages over ACEI/ARB alone in terms of overall response rate and laboratory parameters, the certainty of evidence for these findings is very low, resulting in substantial uncertainty. These findings primarily highlight the weakness and limitations of the current evidence base rather than providing clear recommendations for any specific regimen. There is a pressing need for more rigorous, high-quality RCTs with longer follow-up periods to elucidate the true efficacy and place of these combination regimens in IgAN management.

## Data Availability

The original contributions presented in the study are included in the article/Supplementary Material, further inquiries can be directed to the corresponding authors.

## References

[B1] Brignardello-Petersen R. Bonner A. Alexander P. E. Siemieniuk R. A. Furukawa T. A. Rochwerg B. (2018). Advances in the GRADE approach to rate the certainty in estimates from a network meta-analysis. J. Clin. Epidemiol. 93, 36–44. 10.1016/j.jclinepi.2017.10.005 29051107

[B2] Brignardello-Petersen R. Mustafa R. A. Siemieniuk R. A. C. Murad M. H. Agoritsas T. Izcovich A. (2019). GRADE approach to rate the certainty from a network meta-analysis: addressing incoherence. J. Clin. Epidemiol. 108, 77–85. 10.1016/j.jclinepi.2018.11.025 30529648

[B3] Brown S. J. Bakker C. J. Theis-Mahon N. R. (2022). Retracted publications in pharmacy systematic reviews. J. Med. Libr. Assoc. JMLA 110 (1), 47–55. 10.5195/jmla.2022.1280 35210962 PMC8830339

[B4] Cai Y. P. (2018). Effect analysis of tripterygium glycosides combined with Telmisartan in the treatment of primary IgA nephropathy patients with moderate proteinuria. Henan Med. Res. 27 (20), 3726–3728.

[B5] Cao S. Chen Q. P. (2012). Efficacy of tripterygium glycosides tablets combined with valsartan in the treatment of proteinuria in IgA nephropathy. Pract. Clin. Med. 13 (11), 9–10+36.

[B6] Chen X. L. (2019). Observation on the efficacy of huangkui capsules combined with olmesartan in the treatment of mild to moderate proteinuria in IgA nephropathy. J. Clin. Ration. Drug Use 12 (10), 90–91.

[B7] Chen J. J. Shou D. W. (2015). Effects of huangkui capsules on serum MCP-1, TNF-α and IL-6 levels in patients with IgA nephropathy of spleen Qi deficiency type. Chin. J. Traditional Med. Sci. Technol. 22 (6), 624–625+637.

[B8] Chen D. Chen Y. W. Luo F. M. Gan C. L. (2021). Effect Analysis of Tripterygium Glycosides Tablets Combined with Benazepril in the Treatment of Primary IgA Nephropathy. Chinese Science and Technology Journal Database (Full-text Edition) Medical and Health 11 (14), 429–430.

[B9] Chen M. Zhang P. Li L. Yu Z. Liu N. Wang L. (2022). Efficacy and safety of glycosides of Tripterygium wilfordii combined with renin-angiotensin system in the treatment of IgA nephropathy: a systematic review and meta-analysis. Emerg. Med. Int. 2022, 1–12. 10.1155/2022/5314105 36212998 PMC9546686

[B10] Cheng J. Zhang W. Zhang X. H. He Q. Tao X. J. Chen J. H. (2009). ACEI/ARB therapy for IgA nephropathy: a Meta analysis of randomised controlled trials. Int. J. Clin. Pract. 63 (6), 880–888. 10.1111/j.1742-1241.2009.02038.x 19490198

[B11] Coppo R. (2017). Clinical and histological risk factors for progression of IgA nephropathy: an update in children, young and adult patients. J. Nephrol. 30 (3), 339–346. 10.1007/s40620-016-0360-z 27815919

[B12] Dias S. Welton N. J. Sutton A. J. Caldwell D. M. Lu G. Ades A. E. (2013). Evidence synthesis for decision making 4: inconsistency in networks of evidence based on randomized controlled trials. Med. Decision making an international journal Soc. Med. Decis. Mak. 33 (5), 641–656. 10.1177/0272989X12455847 23804508 PMC3704208

[B13] Egger M. Davey Smith G. Schneider M. Minder C. (1997). Bias in meta-analysis detected by a simple, graphical test. BMJ Clin. Res. Ed. 315 (7109), 629–634. 10.1136/bmj.315.7109.629 9310563 PMC2127453

[B14] Fan Y. Deng C. Yu H. X. (2013). Clinical value of combined application of tripterygium glycosides and fosinopril in the treatment of IgA nephropathy. Guide China Med. 11 (11), 154–155.

[B15] Fan H. L. Wang Y. F. Fu Y. H. (2025). Effect of jinshuibao combined with valsartan on proteinuria and renal function in patients with chronic glomerulonephritis. Chin. J. Mod. Drug Appl. 19 (8), 1–5.

[B16] Fang H. Zhang X. H. Zhang C. J. (2017). Clinical efficacy of tripterygium glycosides combined with cozaar in elderly patients with IgA nephropathy and its effects on the expression of TGF-β1, PAI-1 and VEGF. Chongqing Med. 46 (21), 2937–2939.

[B17] Feng W. (2020). Observation on the efficacy of tripterygium glycosides combined with Telmisartan in the treatment of primary IgA nephropathy with proteinuria. J. Metallurgical Industry Med. 37 (3), 321.

[B18] Gao Y. Guyatt G. Uyeki T. M. Liu M. Chen Y. Zhao Y. (2024). Antivirals for treatment of severe influenza: a systematic review and network meta-analysis of randomised controlled trials. Lancet London, Engl. 404 (10454), 753–763. 10.1016/s0140-6736(24)01307-2 PMC11369965 39181595

[B19] Guan X. D. Wu Z. W. (2005). Clinical observation of huobahuagen tablets combined with irbesartan in the treatment of IgA nephropathy. J. Chin. Integr. Med. 5, 36–39. 10.3736/jcim20050509 16159570

[B20] Guan Y. B. Luo F. Z. Zhao X. F. (2015). Study on the clinical efficacy, safety of tripterygium glycosides combined with benazepril hydrochloride in the treatment of immunoglobulin A nephropathy. Chin. J. Clin. Pharmacol. 10, 787–789.

[B21] Guo B. Q. (2017). Observation on the efficacy of tripterygium glycosides tablets combined with Telmisartan in the treatment of middle-aged, elderly patients with IgA nephropathy. Electron. J. Clin. Med. Literature 4 (70), 13804.

[B22] Guo M. Du Y. L. (2023). Efficacy and mechanism of huobahuagen tablets combined with losartan potassium tablets in the treatment of IgA nephropathy. Pract. Clin. J. Integr. Traditional Chin. West. Med. 23 (7), 21–23+40.

[B23] Guo Y. Q. Xu Y. Y. F. (2019). Observation on the efficacy and safety of huangkui capsules combined with valsartan in IgA nephropathy. Chin. Sci. Technol. J. Database 4 (07), 50‐51.

[B24] Guyatt G. H. Oxman A. D. Vist G. E. Kunz R. Falck-Ytter Y. Alonso-Coello P. (2008). GRADE: an emerging consensus on rating quality of evidence and strength of recommendations. BMJ Clin. Res. Ed. 336 (7650), 924–926. 10.1136/bmj.39489.470347.ad 18436948 PMC2335261

[B25] Han Y. R. Qiu Z. Y. (2010). Clinical study of huangkui capsules combined with benazepril in the treatment of primary IgA nephropathy. Chin. J. Integr. Traditional West. Nephrol. 11 (11), 998–999.

[B26] He L. J. Li B. H. Wu J. Y. Dilinu’er Sun X. H. Gao J. L. (2016). “Clinical observation of Shenyan Kangfu tablets combined with losartan in the treatment of IgA nephropathy,” in paper presented at: the inaugural conference of the digital traditional chinese medicine branch of the international digital medical association and the first digital traditional chinese medicine academic exchange conference 2016. Zhuhai, Guangdong, China.

[B27] Higgins J. P. Jackson D. Barrett J. K. Lu G. Ades A. E. White I. R. (2012). Consistency and inconsistency in network meta-analysis: concepts and models for multi-arm studies. Res. synthesis methods 3 (2), 98–110. 10.1002/jrsm.1044 26062084 PMC4433772

[B28] Higgins J. P. T. Chandler J. (Editors) (2022). Cochrane handbook for systematic reviews of interventions version (London: Cochrane Collaboration).

[B29] Hu J. E. (2021). Effect analysis of tripterygium glycosides combined with irbesartan in the treatment of IgA nephropathy. Jilin Med. J. 42 (11), 2680–2681.

[B30] Huang Q. He J. Q. (2011). Clinical observation of 33 cases of IgA nephropathy treated with bailing capsules combined with benazepril. J. Guiyang Coll. Traditional Chin. Med. 33 (1), 29–31.

[B31] Huang J. Wang L. X. Hou H. J. Hu Q. Q. (2012). Clinical Observation of 25 cases of IgA nephropathy treated with weiyan kangfu tablets combined with fosinopril. Henan Tradit. Chin. Med. 32 (12), 1646–1647.

[B32] Huo Y. Wu W. (2016). Clinical efficacy observation of 31 cases of IgA nephropathy treated with bailing capsules combined with irbesartan. World Chin. Med. 11 (B03), 1270‐1271.

[B33] Hutton B. Salanti G. Caldwell D. M. Chaimani A. Schmid C. H. Cameron C. (2015). The PRISMA extension statement for reporting of systematic reviews incorporating network meta-analyses of health care interventions: checklist and explanations. Ann. Intern. Med. 162 (11), 777–784. 10.7326/m14-2385 26030634

[B34] Jarrick S. Lundberg S. Welander A. Carrero J. J. Höijer J. Bottai M. (2019). Mortality in IgA nephropathy: a nationwide population-based cohort study. J. Am. Soc. Nephrol. 30 (5), 866–876. 10.1681/ASN.2018101017 30971457 PMC6493992

[B35] Kidney Disease: Improving Global Outcomes (KDIGO) CKD Work Group (2024). KDIGO 2024 clinical Practice Guideline for the evaluation and management of chronic kidney disease. Kidney Int. 105 (4), S117–S314. 10.1016/j.kint.2023.10.018 38490803

[B36] König J. Krahn U. Binder H. (2013). Visualizing the flow of evidence in network meta-analysis and characterizing mixed treatment comparisons. Statistics medicine 32 (30), 5414–5429. 10.1002/sim.6001 24123165

[B37] Li P. (2017). Observation on the efficacy of bailing capsules combined with Benazepril in the treatment of IgA nephropathy. Health Med. Res. Pract. 14 (2), 68–69.

[B38] Li Y. F. Huang L. J. (2021). Efficacy and safety of tripterygium glycosides combined with Telmisartan in the treatment of IgA nephropathy. Clin. Res. Pract. 6 (34), 63–65.

[B39] Li M. Y. Piao L. H. (2017). Clinical efficacy of Huangkui capsules combined with Olmesartan Medoxomil in the treatment of mild to moderate proteinuria in IgA nephropathy. China Health Care and Nutr. 27 (35), 186.

[B40] Li L. S. Yan C. P. Zhou Z. H. (2012). Clinical observation of huangkui capsules combined with Olmesartan in the treatment of mild to moderate proteinuria in IgA nephropathy. China Med. Her. 9 (6), 74–75.

[B41] Li Y. Liu H. Yan H. Xiong J. (2023). Research advances on targeted-treg therapies on immune-mediated kidney diseases. Autoimmun. Rev. 22 (2), 103257. 10.1016/j.autrev.2022.103257 36563769

[B42] Li Y. Hou J. Wu X. Liu C. Zhou M. Lin S. (2025). Efficacy of tripterygium glycosides in immune-mediated kidney diseases as a immunomodulation drug in combination with conventional immunosuppressive agents: a systematic review and meta-analysis of randomized controlled trials. Front. Pharmacol. 16, 1525482. 10.3389/fphar.2025.1525482 40717986 PMC12289580

[B43] Liang Y. Zhang L. P. Lu Y. B. Guo W. (2017). Clinical observation of huangkui capsules combined with losartan potassium in the treatment of IgA nephropathy. Shaanxi J. Traditional Chin. Med. 38 (9), 1192–1193.

[B44] Liang Y. Zhang X. L. Liu B. Zhu Q. Shao F. M. (2019). Effects of tripterygium glycosides combined with Irbesartan on efficacy and urinary podocyte Excretion in IgA nephropathy. Chin. General Pract. 22 (12), 1426–1431.

[B45] Lin L. J. Chen X. Q. Wu L. H. Wang Y. Z. Long Z. P. (2014). Clinical observation of Benazepril combined with jinshuibao in the treatment of IgA nephropathy. Guizhou Med. J. 38 (3), 234–235.

[B46] Liu L. Li Q. F. Liu Z. F. (2018). Efficacy Study of bailing capsules combined with Irbesartan in the treatment of IgA nephropathy. World Latest Med. Inf. Abstr. 18 (89), 110–111.

[B47] Lu W. J. (2020). Observation on the clinical efficacy of Huangkui capsules combined with valsartan tablets in the treatment of mild to moderate proteinuria in IgA nephropathy. Med. Forum 24 (16), 2287–2289.

[B48] Lu H. Q. (2021). Efficacy and safety analysis of huangkui capsules combined with dipyridamole tablets, methylprednisolone tablets and Valsartan in the treatment of IgA nephropathy patients. Harbin Med. J. 41 (6), 128–129.

[B49] Lu X. M. Tang X. L. Qin D. Y. (2016). Tripterygium glycosides combined with RAS inhibitors in the treatment of CKD2∼3 stage IgA nephropathy. J. Pract. Med. 32 (1), 137–139.

[B50] Luo L. Liu Z. Q. Fan Y. J. (2023). Bailing Capsules assisted Valsartan in the treatment of IgA nephropathy and its effects on renal function, Th1/Th2 Drift, urinary PCX and B7-1 levels. Guangzhou Med. J. 54 (2), 46–49+60.

[B51] Ma F. Zhao H. Y. (2016). Observation on the clinical efficacy of Huangkui capsules combined with Olmesartan Medoxomil in the treatment of mild to moderate proteinuria in IgA nephropathy. China Pract. Med. 11 (27), 195–196.

[B52] Pei S. Li Y. (2021). Huangkui capsule in combination with leflunomide improves immunoglobulin A nephropathy by inhibiting the TGF-β1/Smad3 signaling pathway. Clin. Sao Paulo, Braz. 76, e2904. 10.6061/clinics/2021/e2904 34909911 PMC8614623

[B53] Peng T. Yang X. D. Li D. R. Guo L. Xia Q. Hu Z. (2010). Efficacy observation of huangkui capsules combined with Valsartan the treatment of IgA nephropathy. Chin. J. Integr. Traditional West. Nephrol. 11 (8), 723–724.

[B54] Puhan M. A. Schünemann H. J. Murad M. H. Brignardello-Petersen R. Singh J. A. (2014). A GRADE working group approach for rating the quality of treatment effect estimates from network meta-analysis. BMJ Clin. Res. Ed. 349, g5630. 10.1136/bmj.g5630 25252733

[B55] Schena F. P. Nistor I. (2018). Epidemiology of IgA nephropathy: a global perspective. Seminars Nephrol. 38 (5), 435–442. 10.1016/j.semnephrol.2018.05.013 30177015

[B56] Shen S. J. Hu Z. X. Wang S. M. Li Q. H. (2009). Efficacy observation of tripterygium glycosides tablets combined with Benazepril in the treatment of IgA nephropathy. Chin. J. Integr. Traditional West. Nephrol. 10 (2), 154–155.

[B57] Shi Y. J. Liu Z. J. H. Zhong W. Q. Huang Z. L. (2005). Clinical study of integrated traditional Chinese and Western medicine quadruple therapy in the treatment of nephrotic syndrome type IgA nephropathy. Chin. J. Integr. Traditional West. Nephrol. 1, 20–22.

[B58] Shi F. Hou L. H. Liu X. (2015). Efficacy observation of 20 cases of IgA nephropathy treated with shenyan kangfu tablets combined with losartan potassium. Hunan J. Traditional Chin. Med. 31 (8), 50–51.

[B59] Sterne J. A. C. Savović J. Page M. J. Elbers R. G. Blencowe N. S. Boutron I. (2019). RoB 2: a revised tool for assessing risk of bias in randomised trials. BMJ Clin. Res. Ed. 366, l4898. 10.1136/bmj.l4898 31462531

[B60] Su B. F. Zhang H. D. (2014). Efficacy observation of huangkui capsules combined with Irbesartan in the treatment of IgA nephropathy. Chin. J. Prev. Control Chronic Dis. 22 (5), 585–586.

[B61] Sun X. H. (2015). Observation on the clinical efficacy of Jinshuibao capsules combined with routine Western medicine in the treatment of primary IgA nephropathy. China Mod. Med. 1, 96–98.

[B62] Tang Y. (2018). Clinical efficacy of Huangkui capsules combined with Irbesartan in the treatment of IgA nephropathy. J. Clin. Ration. Drug Use 11 (6), 16–17.

[B63] Tang W. G. Xu M. Lu J. K. Ma G. X. (2009). Clinical study of huangkui capsules combined with lisinopril in the treatment of IgA nephropathy. Pract. Clin. J. Integr. Traditional Chin. West. Med. 9 (3), 34–35.

[B64] Wang X. Y. (2016). Clinical efficacy analysis of tripterygium glycosides tablets combined with benazepril hydrochloride in the treatment of primary IgA nephropathy. China Pract. Med. 11 (14), 130–131.

[B65] Wang Y. H. (2020). Clinical efficacy of tripterygium glycosides tablets combined with Olmesartan in the treatment of primary IgA nephropathy patients. Guide China Med. 18 (29), 132–133.

[B66] Wang K. M. Lai C. S. S. (2005). Study on huobahuagen in the treatment of IgA nephropathy. Mod. J. Integr. Traditional Chin. West. Med. 14 (14), 1819‐1820.

[B67] Wang D. Liu X. J. (2018). Study on the clinical efficacy and safety of tripterygium glycosides combined with benazepril hydrochloride in the treatment of immunoglobulin A nephropathy. Chin. J. Mod. Drug Appl. 12 (4), 109–110.

[B68] Wang Y. H. Wang B. D. Xu D. M. (2024). Effect of bailing capsule on urine osmotic pressure, urinary protein and levels of PCX, B7-1, β_2_-MG and NAG in patients with IgA nephropathy. Syst. Med. 9 (12), 1–4+9.

[B69] Wang S. Yang L. Huang P. Zheng J. Wang Q. Liu H. F. (2025). Dapagliflozin reduces proteinuria in IgA nephropathy patients receiving immunosuppressive therapy. Ren. Fail. 47 (1), 2555998. 10.1080/0886022x.2025.2555998 40926407 PMC12424146

[B70] Wei J. W. (2019). Efficacy observation of tripterygium glycosides combined with Irbesartan in the treatment of IgA nephropathy. J. Pract. Traditional Chin. Med. 35 (2), 187.

[B71] Wei L. Zheng Y. Wang Y. (2019). Effects of benazepril combined with tripterygium glycosides on cellular immune function and serum VEGF, ET-1 levels in IgA nephropathy. Clin. Misdiagnosis and Mistherapy 32 (7), 25–29.

[B72] Xiang Q. Song E. F. Liu H. Y. (2014). Observation on the efficacy of tripterygium glycosides tablets combined with Telmisartan in the treatment of middle-aged and elderly patients with IgA nephropathy. World J. Integr. Traditional West. Med. 9 (7), 756–758.

[B73] Xu L. (2020). Efficacy of Losartan combined with tripterygium glycosides in the treatment of IgA nephropathy and its effect on immune function. J. Med. Theory Pract. 33 (23), 3927–3928.

[B74] Xu K. Li Q. Bu H. X. (2009). Analysis of 80 cases of IgA Nephropathy treated with huangkui capsules combined with lotensin. Chin. J. Med. Guide 11 (9), 1519–1520.

[B75] Yang J. H. (2010). Efficacy observation of huangkui capsules combined with Valsartan in the treatment of IgA nephropathy. Chin. J. Integr. Traditional West. Nephrol. 11 (9), 831–832.

[B76] Yang L. Chen Q. K. (2010). Efficacy observation of 21 cases of IgA Nephropathy treated with benazepril hydrochloride, dipyridamole combined with huangkui capsules. J. Nanchang Univ. Med. Ed. 50 (6), 69–70.

[B77] Yang B. Liu Q. Zhu Y. Xie H. P. Zhao H. S. Guan L. (2013). Clinical study of irbesartan combined with shenyan kangfu tablets in the treatment of proteinuria in IgA nephropathy. J. Clin. Chin. Med. 5 (1), 12–13.

[B78] Yang Z. M. Shen S. Z. Hu Y. Y. Cai J. Y. Sun L. Y. Xie Q. H. (2014). Clinical study of tripterygium glycosides combined with benazepril in the treatment of immunoglobulin A nephropathy. Chin. J. Clin. Pharmacol. 11, 994–995.

[B79] Yang Z. M. Shen S. Z. Hu Y. Y. Cai J. Y. Sun L. Y. Xie Q. H. (2016). Short-term efficacy observation of benazepril combined with shenyan kangfu tablets in the treatment of IgA nephropathy. Chin. J. Integr. Traditional West. Nephrol. 17 (5), 442–443.

[B80] Yin Y. (2023). Analysis of the effect of combined use of huangkui capsules and Benazepril on the long-term prognosis of IgA nephropathy patients. J. Shanxi Health Vocat. Coll. 33 (2), 67–69.

[B81] Yu T. (2016). Study on the clinical efficacy of tripterygium glycosides combined with Benazepril in the treatment of immunoglobulin A nephropathy. Guide China Med. 14 (35), 141.

[B82] Yu X. F. Huang Z. D. (2012). Fosinopril combined with tripterygium glycosides in the treatment of IgA nephropathy with moderate proteinuria. Chin. J. Integr. Traditional West. Nephrol. 13 (5), 438–439.

[B83] Yu X. L. Zhu G. N. (2015). Effects of bailing capsules combined with Benazepril on urinary protein and serum creatinine in patients with IgA nephropathy. J. New Chin. Med. 47 (3), 83–84.

[B84] Yuan L. Cai K. Zou Y. (2023). Clinical efficacy of Huangkui capsule plus methylprednisolone for Immunoglobulin A nephropathy and its effect on renal function and serum inflammatory factors. Evidence-based complementary alternative medicine eCAM. 2023, 3020033. 10.1155/2023/3020033 36865740 PMC9974270

[B85] Zeng C. (2021). Evaluation of the efficacy of bailing capsule combined with Benazepril in the treatment of IgA nephropathy. Self Healthc. 14, 72–73.

[B86] Zhang W. (2014). Clinical analysis of huangkui capsules combined with Telmisartan in the treatment of mild to moderate proteinuria in IgA nephropathy. Contemp. Med. 20 (25), 133–134.

[B87] Zhang C. Z. (2021). Clinical analysis of huangkui capsules combined with Irbesartan in the treatment of IgA nephropathy. Chin. J. Integr. Traditional West. Nephrol. 22 (12), 1099–1100.

[B88] Zhang N. Xia T. (2010). Clinical observation and analysis of 102 cases of IgA Nephropathy treated with huangkui capsules combined with Losartan. Chin. J. Integr. Traditional West. Nephrol. 11 (12), 1108.

[B89] Zhang N. Xia T. (2011). “Clinical observation and analysis of 62 cases of IgA Nephropathy treated with huangkui capsules combined with Losartan,” in Proceedings of the 9th National Nephrology Academic Conference of the Geriatrics Branch of the Chinese Medical Association. Wuhan, 94–95.

[B90] Zhang N. Yang H. T. (2016). Effects of Valsartan combined with bailing capsules on urinary protein, urinary osmolality and clinical safety in patients with IgA nephropathy. West. J. Traditional Chin. Med. 29 (10), 116–118.

[B91] Zhang Y. X. Peng T. Guo L. Xia Q. (2010). Efficacy observation of jinshuibao combined with valsartan in the treatment of IgA nephropathy. Pract. J. Med. and Pharm. 27 (5), 413–414.

[B92] Zhang X. Z. Wang D. He L. Q. Liu K. Shen B. (2013). Clinical observation of shenyan kangfu tablets combined with losartan potassium in the treatment of primary IgA nephropathy (Qi-Yin deficiency syndrome). J. Clin. Nephrol. 13 (2), 83–86.

[B93] Zhang C. Wang M. L. Zhang Y. (2014). Clinical Study of huangkui capsules combined with losartan in the treatment of mild to moderate proteinuria in IgA nephropathy. China Pharm. 23 (18), 112–114.

[B94] Zhang Y. M. Lv J. C. Wong M. G. Zhang H. Perkovic V. (2023). Glucocorticoids for IgA nephropathy-pro. Kidney Int. 103 (4), 666–669. 10.1016/j.kint.2023.01.018 36948768

[B95] Zhao J. (2013). Effect of benazepril combined with huangkui capsules on urinary MCP-1 in IgA nephropathy patients with mild to moderate proteinuria. Chin. J. Pract. Med. 40 (23), 15‐17.

[B96] Zhou H. X. (2002). Efficacy observation of bailing capsules in the treatment of hematuria in IgA nephropathy. Traditional Chin. Med. Res. 4, 26–27.

[B97] Zhou H. L. Zou X. R. Wang X. Q. (2012). Clinical observation of huangkui capsules combined with benazepril hydrochloride in the treatment of IgA nephropathy. J. Clin. Nephrol. 12 (9), 400.

