# Supplementary material for: Which commercial Chinese polyherbal preparations combined with ACEI/ARB is effective and safe for IgA nephropathy? A systematic review and network meta-analysis
Source: Front Pharmacol. 2026 Jun 24;17:1755995. doi: 10.3389/fphar.2026.1755995 (PMC13342400; doi:10.3389/fphar.2026.1755995)

Appendix Material for

**Which commercial Chinese polyherbal preparations combined with ACEI/ARB is effective and safe for IgA nephropathy? A systematic review and network meta-analysis**

Appendix 1: Search strategy for databases

Appendix 2. Information of included CCPPs

Appendix 3: Risk of bias for eligible studies

Appendix 4: Assessment of between-study heterogeneity

Appendix 5: Assessment of inconsistency

Appendix 6: GRADE summary of findings for outcomes

Appendix 7: SUCRA plots

Appendix 8: Assessment of SUCRA

Appendix 9. Detailed adverse drug reactions by intervention

Appendix 10: The results of sensitivity analysis

Appendix 1. Search strategy for databases

**PubMed**

#1 iga nephropath *[Title/Abstract] OR Immunoglobulin a Nephropathy *[Title/Abstract] OR immunoglobulin a nephropathy *[Title/Abstract] OR iga glomeruloneph *[Title/Abstract] OR berger disease *[Title/Abstract] OR IgAGN *[Title/Abstract] OR igAN *[Title/Abstract]

#2 "tripterygium glycosides" [Supplementary Concept] OR tripterygium glycosides *[Title/Abstract]

#3 "bailing" [Supplementary Concept] OR bailing *[Title/Abstract]

#4 "jinshuibao" [Supplementary Concept] OR jinshuibao *[Title/Abstract]

#5 "huobahuagen" [Supplementary Concept] OR huobahuagen *[Title/Abstract]

#6 "huangkui" [Supplementary Concept] OR huangkui *[Title/Abstract]

#7 "shenyankangfu" [Supplementary Concept] OR shenyankangfu *[Title/Abstract]

#8 #2 OR #3 OR #4 OR #5 OR #6 OR #7

#9 "Randomized Controlled Trial"[Publication Type] OR "Controlled Clinical Trial"[Publication Type] OR "Clinical Trials as Topic"[Mesh] OR randomized [Title/Abstract] OR randomized [Title/Abstract] OR randomly [Title/Abstract] OR placebo [Title/Abstract] OR trial [Title]

#10 #1 AND #8 AND #9

**Embase**

#1 'iga nephropath':ab,ti OR 'Immunoglobulin a Nephropathy':ab,ti OR 'immunoglobulin a nephropathy':ab,ti OR 'iga glomeruloneph':ab,ti OR 'berger disease':ab,ti OR 'IgAGN':ab,ti OR 'igAN':ab,ti

#2 bailing:ab,ti

#3 jinshuibao:ab,ti

#4 huobahuagen:ab,ti

#5 tripterygium glycosides:ab,ti

#6 huangkui:ab,ti

#7 shenyankangfu:ab,ti

#8 #2 OR #3 OR #4 OR #5 OR #6 OR #7

#9 'randomized controlled trial':it OR 'controlled clinical trial':it OR 'clinical trials as topic'/exp/mj OR randomized:ab,ti OR randomly:ab,ti OR placebo:ab,ti OR trial:ti

#10 #1 AND #8 AND #9

**The Cochrane Central Register of Controlled Trials**

#1 (iga nephropathy):ti,ab,kw OR (Immunoglobulin a Nephropathy):ti,ab,kw OR (immunoglobulin a nephropathy):ti,ab,kw OR (iga glomeruloneph):ti,ab,kw OR (berger disease):ti,ab,kw OR (IgAGN):ti,ab,kw OR (igAN):ti,ab,kw

#2 (bailing):ti,ab,kw

#3 (jinshuibao):ti,ab,kw

#4 (huobahuagen):ti,ab,kw

#5 (tripterygium glycosides):ti,ab,kw

#6 (huangkui):ti,ab,kw

#7 (shenyankangfu):ti,ab,kw

#8 #2 OR #3 OR #4 OR #5 OR #6 OR #7

#9 #1 AND #8

**The ClinicalTrials.gov**

Condition or disease: iga nephropathy

Intervention/Treatment: bailing or jinshuibao or huobahuagen or tripterygium glycosides or huangkui or shenyankangfu

**CNKI (中国知网)**

#1 ((SU='肾炎' AND SU='IgA') OR (SU='IgA 肾病' OR SU='IgA 肾脏病' OR SU='Berger病' OR SU='IgA 肾炎' OR SU='IgAN'))

#2 ((SU='中成药' OR SU='成药') OR (TI='中成药' OR TI='成药'))

#3 (SU='百令胶囊' OR SU='百令片' OR SU='百令') OR (TI='百令胶囊' OR TI='百令片' OR TI='百令')

#4 (SU='金水宝胶囊' OR SU='金水宝片' OR SU='金水宝') OR (TI='金水宝胶囊' OR TI='金水宝片' OR TI='金水宝')

#5 (SU='火把花根片') OR (TI='火把花根片')

#6 (SU='雷公藤多苷片' OR SU='雷公藤多苷') OR (TI='雷公藤多苷片' OR TI='雷公藤多苷')

#7 (SU='黄葵胶囊' OR SU='黄葵') OR (TI='黄葵胶囊' OR TI='黄葵')

#8 SU='肾炎康复片' OR TI='肾炎康复片'

#9 #2 OR #3 OR #4 OR #5 OR #6 OR #7 OR #8

#10 #1 AND #9

**WanFang (万方医学)**

#1 ((主题: 肾炎 AND 主题: IgA) OR (主题: IgA 肾病 OR 主题: IgA 肾脏病 OR 主题: Berger病 OR 主题: IgA 肾炎 OR 主题: IgAN))

#2 ((主题: 中成药 OR 主题: 成药) OR (题名或关键词: 中成药 OR 题名或关键词: 成药))

#3 (主题: 百令胶囊 OR 主题: 百令片 OR 主题: 百令) OR (题名或关键词: 百令胶囊 OR 题名或关键词: 百令片 OR 题名或关键词: 百令)

#4 (主题: 金水宝胶囊 OR 主题: 金水宝片 OR 主题: 金水宝) OR (题名或关键词: 金水宝胶囊 OR 题名或关键词: 金水宝片 OR 题名或关键词: 金水宝)

#5 (主题: 火把花根片) OR (题名或关键词: 火把花根片)

#6 (主题: 雷公藤多苷片 OR 主题: 雷公藤多苷) OR (题名或关键词: 雷公藤多苷片 OR 题名或关键词: 雷公藤多苷)

#7 (主题: 黄葵胶囊 OR 主题: 黄葵) OR (题名或关键词: 黄葵胶囊 OR 题名或关键词: 黄葵)

#8 主题: 肾炎康复片 OR 题名或关键词: 肾炎康复片

#9 OR #3 OR #4 OR #5 OR #6 OR #7 OR #8

#10 #1 AND #9

**VIP (维普数据库)**

#1 ((M=肾炎 AND M=IgA) OR (M=IgA 肾病 OR M=IgA 肾脏病 OR M=Berger病 OR M=IgA 肾炎 OR M=IgAN))

#2 (M=中成药 OR M=成药)

#3 (M=百令胶囊 OR M=百令片 OR M=百令)

#4 (M=金水宝胶囊 OR M=金水宝片 OR M=金水宝)

#5 M=火把花根片

#6 (M=雷公藤多苷片 OR M=雷公藤多苷)

#7 (M=黄葵胶囊 OR M=黄葵)

#8 M=肾炎康复片

#9 2 OR #3 OR #4 OR #5 OR #6 OR #7 OR #8

#10 1 AND #9

**SinoMed (中国生物医学数据库)**

#1 "IgA 肾病"[不加权:扩展] OR "IgA 肾脏病"[不加权:扩展] OR "Berger病"[不加权:扩展] OR "IgA 肾炎"[不加权:扩展] OR "IgAN"[不加权:扩展]

#2 "中成药"[常用字段:智能] OR "成药"[常用字段:智能]

#3 "百令胶囊"[常用字段:智能] OR "百令片"[常用字段:智能] OR "百令"[常用字段:智能]

#4 "金水宝胶囊"[常用字段:智能] OR "金水宝片"[常用字段:智能] OR "金水宝"[常用字段:智能]

#5 "火把花根片"[常用字段:智能]

#6 "雷公藤多苷片"[常用字段:智能] OR "雷公藤多苷"[常用字段:智能]

#7 "黄葵胶囊"[常用字段:智能] OR "黄葵"[常用字段:智能]

#8 "肾炎康复片"[常用字段:智能]

#9 #2 OR #3 OR #4 OR #5 OR #6 OR #7 OR #8

#10 #1 AND #9

Appendix 2. Information of included CCPPs

| Brand name | Dosage form | Source of composition information | Medicinal ingredients | Taxonomically Validated Full Species Name (with Authority, Family & Pharmacopoeial Drug Name) | Single‑herb or polyherbal | Consistency of composition across included studies |
| --- | --- | --- | --- | --- | --- | --- |
| BL | capsule | NMPA official data, official package insert, Chinese Pharmacopoeia | Fermented Cordyceps sinensis mycelial powder | Ophiocordyceps sinensis (Berk.) G.H. Sung, J.M. Sung, Hywel-Jones & Spatafora [Ophiocordycipitaceae; Cordyceps] | Single‑herb | Consistent; all included studies used preparations with identical fermented Cordyceps sinensis mycelial powder |
| JSB | capsule | NMPA official data, official package insert, Chinese Pharmacopoeia | Fermented Cordyceps militaris mycelial powder | Cordyceps militaris (L.) Fr. [Cordycipitaceae; Cordyceps Militaris] | Single‑herb | Consistent; all included studies used preparations with identical fermented Cordyceps militaris mycelial powder |
| HBHG | tablet | NMPA official data, official package insert, original trials | Tripterygium hypoglaucum extract | Tripterygium hypoglaucum (H. Lév.) Hutch. [Celastraceae; Tripterygii Hypoglauci Radix] | Single‑herb | Consistent; all preparations contained standardized Tripterygium hypoglaucum extract |
| TG | tablet | NMPA official data, Chinese Pharmacopoeia, original trials | Tripterygium wilfordii root glycosides extract | Tripterygium wilfordii Hook. f. [Celastraceae; Tripterygii Wilfordii Radix] | Single‑herb | Consistent; all preparations contained standardized tripterygium glycosides extract |
| SYKF | tablet | NMPA official data, official package insert, Chinese Pharmacopoeia | Panax quinquefolius, Panax ginseng, Rehmannia glutinosa, Eucommia ulmoides (stir‑fried), Dioscorea polystachya, Hedyotis diffusa, Glycine max, Smilax glabra, Leonurus japonicus, Salvia miltiorrhiza, Alisma orientale, Imperata cylindrica, Platycodon grandiflorus | 1. Panax quinquefolius L. [Araliaceae; Panacis Quinquefolii Radix] 2. Panax ginseng C. A. Mey. [Araliaceae; Ginseng Radix et Rhizoma] 3. Rehmannia glutinosa (Gaertn.) DC. [Orobanchaceae; Rehmanniae Radix] 4. Eucommia ulmoides Oliv. [Eucommiaceae; Eucommiae Cortex] 5. Dioscorea polystachya Turcz. [Dioscoreaceae; Dioscoreae Rhizoma] 6. Hedyotis diffusa Willd. [Rubiaceae; Hedyotidis Diffusae Herba] 7. Glycine max (L.) Merr. [Fabaceae; Sojae Semen Nigrum] 8. Smilax glabra Roxb. [Smilacaceae; Smilacis Glabrae Rhizoma] 9. Leonurus japonicus Houtt. [Lamiaceae; Leonuri Herba] 10. Salvia miltiorrhiza Bunge [Lamiaceae; Salviae Miltiorrhizae Radix et Rhizoma] 11. Alisma orientale (Sam.) Juz. [Alismataceae; Alismatis Rhizoma] 12. Imperata cylindrica (L.) Raeusch. [Poaceae; Imperatae Rhizoma] 13. Platycodon grandiflorus (Jacq.) A. DC. [Campanulaceae; Platycodonis Radix] | Polyherbal | Consistent; fixed standard prescription used across all included studies |
| HK | capsule | NMPA official data, official package insert, Chinese Pharmacopoeia | Abelmoschus manihot flower extract | Abelmoschus manihot (L.) Medik. [Malvaceae; Abelmoschi Manihotis Flos] | Single‑herb | Consistent; all preparations contained standardized Abelmoschus manihot flower extract |

Notes: NMPA, National Medical Products Administration; CCPPs, commercial Chinese polyherbal preparations; BL, Bailing capsule; HBHG, Huobahuagen Tablet; HK, Huangkui capsule; TG, Tripterygium glycosides; JSB, Jinshuibao capsule; SYKF, Shenyan Kangfu Tablet.

Appendix 3. Risk of bias for eligible studies

| Study | Bias arising from the randomization process | Bias due to deviations from the intended intervention | Bias due to missing outcome data | Bias in measurement of the outcome | Bias in selection of the reported results | Other risk of bias | Overall judgement |
| --- | --- | --- | --- | --- | --- | --- | --- |
| Zengcong 2021 | Probably Low | Probably High | Low | Probably High | Probably Low | Probably Low | Probably High |
| Liulin 2018 | Probably Low | Probably High | Low | Probably High | Probably Low | Probably Low | Probably High |
| Luoli 2023 | Probably Low | High | Low | Probably High | Probably Low | Probably Low | Probably High |
| Liping 2017 | Probably Low | Probably Low | Low | Probably Low | Probably Low | Probably Low | Probably Low |
| Yuxuelei 2015 | Probably Low | Probably Low | Low | Probably Low | Low | Probably Low | Probably Low |
| Huangqin 2011 | Probably High | Probably High | Low | Probably Low | Low | Probably Low | Probably High |
| Huoyan 2016 | Probably High | Probably High | Low | Probably Low | Low | Probably Low | Probably High |
| Zhouhongxing 2002 | Probably High | Probably High | Probably Low | Probably High | Low | Probably Low | Probably High |
| Zhangnan 2016 | Probably High | Probably High | Low | Probably High | Probably Low | Probably Low | Probably High |
| Guanxiaodong 2005 | Probably High | Probably High | Low | Probably Low | Probably Low | Probably Low | Probably High |
| Guomin 2023 | Low | Probably Low | Low | Probably Low | Low | Probably Low | Probably Low |
| Wangkunming 2005 | Probably Low | Probably Low | Low | Probably Low | Low | Probably Low | Probably Low |
| Zhaojian 2013 | Probably Low | Probably Low | Low | Probably Low | Low | Probably Low | Probably Low |
| Mafei 2016 | Probably High | Probably High | Low | Probably Low | Probably Low | Probably Low | Probably High |
| Limingyu 2017 | Probably Low | Probably High | Low | Probably Low | Probably Low | Probably Low | Probably High |
| Chenjianjun 2015 | Probably Low | Probably Low | Low | Probably Low | Low | Probably Low | Probably Low |
| Chenxuelian 2019 | Low | Probably Low | Low | Low | Low | Probably Low | Probably Low |
| Liliusheng 2012 | Low | Probably Low | Low | Low | Low | Probably Low | Probably Low |
| Hanyarong 2010 | High | High | Low | Probably Low | Low | Probably Low | High |
| Supofeng 2014 | High | High | Low | Probably Low | Low | Probably Low | High |
| Tangyi 2018 | Probably Low | Probably High | Low | Probably Low | Low | Probably Low | Probably High |
| Zhangchunzhao 2021 | Probably Low | Probably Low | Low | Probably Low | Low | Probably Low | Probably Low |
| Tangweigang 2009 | Probably Low | Probably Low | Low | Probably Low | Low | Probably Low | Probably Low |
| Xuke 2009 | Probably High | Probably High | Low | Probably High | Probably Low | Probably Low | Probably High |
| Liangyan 2017 | High | High | Low | Probably High | Probably Low | Probably Low | High |
| Zhangning 2011 | High | High | Low | Probably High | Probably Low | Probably Low | High |
| Zhangning 2010 | Probably High | Probably High | Low | Probably Low | Probably Low | Probably Low | Probably High |
| Zhangchen 2014 | Probably Low | Probably High | Low | Probably Low | Low | Probably Low | Probably High |
| Luhuiqin 2021 | Low | Probably Low | Low | Probably Low | Low | Probably Low | Probably Low |
| Zhangwei 2014 | High | High | Low | Probably Low | Probably Low | Probably Low | High |
| Guoyuqiang 2019 | High | High | Low | Probably Low | Probably Low | Probably Low | High |
| Luwenjuan 2020 | Probably Low | Probably Low | Low | Probably Low | Low | Probably Low | Probably Low |
| Yangjuhong 2010 | High | High | Low | Probably Low | Probably Low | Probably Low | High |
| Pengtao 2010 | Probably High | Probably High | Low | Probably Low | Low | Probably Low | Probably High |
| Zhouhuilan 2012 | Probably High | High | Probably High | Probably High | Probably Low | Probably Low | High |
| Yinyong 2023 | Low | Probably Low | Low | Probably Low | Low | Probably Low | Probably Low |
| Yangliu 2010 | Probably Low | Probably Low | Low | Probably Low | Low | Probably Low | Probably Low |
| Linlijuan 2014 | Probably Low | Probably Low | Low | Probably Low | Low | Probably Low | Probably Low |
| Sunxuehui 2015 | High | High | Low | Probably Low | Probably Low | Probably Low | High |
| Zhangyongxiu 2010 | Probably High | Probably High | Low | Probably Low | Probably Low | Probably Low | Probably High |
| Yangzhongmin 2016 | Probably Low | Probably Low | Low | Probably Low | Low | Probably Low | Probably Low |
| Yangpo 2013 | Probably High | Probably High | Low | Probably Low | Probably Low | Probably Low | Probably High |
| Shifeng 2015 | Probably Low | Probably High | Low | Probably Low | Low | Probably Low | Probably High |
| Zhangxinzhi 2013 | High | High | Low | Probably Low | Probably Low | Probably Low | High |
| Helijuan 2016 | Probably High | Probably High | Low | Probably Low | Low | Probably Low | Probably High |
| Huangjing 2012 | High | High | Probably Low | Probably High | Probably Low | Probably Low | High |
| Weilan 2019 | Probably Low | Probably Low | Low | Probably Low | Low | Probably Low | Probably Low |
| Yuxufeng 2012 | High | High | Low | Probably Low | Probably Low | Probably Low | High |
| Fanye 2013 | Low | Probably Low | Low | Probably Low | Probably Low | Probably Low | Probably Low |
| Luxiaomei 2016 | Low | Low | Low | Low | Low | Probably Low | Low |
| Yutao 2016 | Probably Low | Probably Low | Low | Probably Low | Probably Low | Probably Low | Probably Low |
| Yangzhongmin 2014 | Low | Probably Low | Low | Probably Low | Low | Probably Low | Probably Low |
| Liangyan 2019 | High | High | Low | Probably Low | Probably Low | Probably Low | High |
| Hujiane 2021 | Probably Low | Probably Low | Low | Probably Low | Probably Low | Probably Low | Probably Low |
| Fanghui 2017 | Low | Probably Low | Low | Probably Low | Probably Low | Probably Low | Probably Low |
| Liyanfeng 2021 | Low | Low | Low | Low | Low | Probably Low | Low |
| Fengwei 2020 | Probably Low | Probably Low | Low | Probably Low | Low | Probably Low | Probably Low |
| Caiyuping 2018 | Low | Probably Low | Low | Probably Low | Low | Probably Low | Probably Low |
| Guanyibiao 2015 | Low | Probably Low | Low | Low | Low | Probably Low | Probably Low |
| Wangdan 2018 | Low | Probably Low | Low | Probably Low | Low | Probably Low | Probably Low |
| Weijiwei 2019 | Probably Low | Probably Low | Low | Probably Low | Probably Low | Probably Low | Probably Low |
| Wangyanhua 2020 | Probably Low | Probably Low | Low | Probably Low | Probably Low | Probably Low | Probably Low |
| Chendu 2021 | Low | Probably Low | Low | Probably Low | Low | Probably Low | Probably Low |
| Shenshuijuan 2009 | High | High | Low | Probably Low | Probably Low | Probably Low | High |
| Guobaoqin 2017 | Low | Probably Low | Low | Probably Low | Low | Probably Low | Probably Low |
| Xiangqiong 2014 | Low | Probably Low | Low | Probably Low | Low | Probably Low | Probably Low |
| Caoshan 2012 | High | High | Low | Probably Low | Probably Low | Probably Low | High |
| Wangxiaoyan 2016 | Probably Low | Probably Low | Low | Probably Low | Low | Probably Low | Probably Low |
| Xulei 2020 | Probably Low | Probably Low | Low | Probably Low | Low | Probably Low | Probably Low |
| Shiyongjun 2005 | High | High | Low | Probably High | Probably Low | Probably Low | High |

Appendix 4. Assessment of between-study heterogeneity

| Outcome | Comparison | No. study | I^2^ (%) |
| --- | --- | --- | --- |
| Overall response rate | BL+ACEI/ARB vs. ACEI/ARB | 6 | 0 |
|  | HBHG+ACEI/ARB vs. ACEI/ARB | 2 | 0 |
|  | HK+ACEI/ARB vs. ACEI/ARB | 18 | 17.5 |
|  | JSB+ACEI/ARB vs. ACEI/ARB | 1 | NA |
|  | SYKF+ACEI/ARB vs. ACEI/ARB | 4 | 0 |
|  | TG+ACEI/ARB vs. ACEI/ARB | 20 | 33.3 |
| 24-hour urinary protein quantity | BL+ACEI/ARB vs. ACEI/ARB | 7 | 87.3 |
|  | HBHG+ACEI/ARB vs. ACEI/ARB | 2 | 45.2 |
|  | HK+ACEI/ARB vs. ACEI/ARB | 19 | 94.1 |
|  | JSB+ACEI/ARB vs. ACEI/ARB | 3 | 98.5 |
|  | SYKF+ACEI/ARB vs. ACEI/ARB | 6 | 92.8 |
|  | TG+ACEI/ARB vs. ACEI/ARB | 15 | 96.3 |
| Serum creatinine | BL+ACEI/ARB vs. ACEI/ARB | 5 | 75.3 |
|  | HBHG+ACEI/ARB vs. ACEI/ARB | 3 | 92.8 |
|  | HK+ACEI/ARB vs. ACEI/ARB | 11 | 89.5 |
|  | JSB+ACEI/ARB vs. ACEI/ARB | 2 | 85.2 |
|  | SYKF+ACEI/ARB vs. ACEI/ARB | 5 | 88.4 |
|  | TG+ACEI/ARB vs. ACEI/ARB | 14 | 91.2 |
| Blood urea nitrogen | BL+ACEI/ARB vs. ACEI/ARB | 3 | 92.5 |
|  | HBHG+ACEI/ARB vs. ACEI/ARB | 2 | 0 |
|  | HK+ACEI/ARB vs. ACEI/ARB | 6 | 89.3 |
|  | SYKF+ACEI/ARB vs. ACEI/ARB | 2 | 85.7 |
|  | TG+ACEI/ARB vs. ACEI/ARB | 7 | 96.8 |
| Hemoglobin | BL+ACEI/ARB vs. ACEI/ARB | 2 | 0 |
|  | HBHG+ACEI/ARB vs. ACEI/ARB | 1 | NA |
|  | HK+ACEI/ARB vs. ACEI/ARB | 12 | 85.3 |
|  | JSB+ACEI/ARB vs. ACEI/ARB | 2 | 69.8 |
|  | SYKF+ACEI/ARB vs. ACEI/ARB | 2 | 0 |
|  | TG+ACEI/ARB vs. ACEI/ARB | 10 | 90.7 |
| Adverse drug reactions | BL+ACEI/ARB vs. ACEI/ARB | 2 | 0 |
|  | HBHG+ACEI/ARB vs. ACEI/ARB | 1 | NA |
|  | HK+ACEI/ARB vs. ACEI/ARB | 1 | NA |
|  | JSB+ACEI/ARB vs. ACEI/ARB | 10 | 52.1 |
|  | TG+ACEI/ARB vs. ACEI/ARB | 15 | 66.3 |

Appendix 5. Assessment of inconsistency

| Outcome | △DIC | P |
| --- | --- | --- |
| Overall response rate | 0.6 | ＞0.05 |
| 24-hour urinary protein quantity | 0.2 | ＞0.05 |
| Serum creatinine | 0.7 | ＞0.05 |
| Blood urea nitrogen | 0.3 | ＞0.05 |
| Hemoglobin | 0.5 | ＞0.05 |
| Adverse drug reactions | 0.3 | ＞0.05 |

Appendix 6. GRADE summary of findings for outcomes

6.1. GRADE summary of findings for overall response rate for different comparisons

| Comparations | Study results and measurements | Certainty in effect estimates |
| --- | --- | --- |
|  |  |  |
| BL+ACEI/ARB vs. ACEI/ARB | Odds Ratio: 3.27 (95%CI: 2.21 to 4.84) Based on data from 372 participants in six studies | Moderate† |
| HBHG+ACEI/ARB vs. ACEI/ARB | Odds Ratio: 2.49 (95%CI: 1.66 to 3.73) Based on data from 172 participants in two studies | Low†‡ |
| HK+ACEI/ARB vs. ACEI/ARB | Odds Ratio: 3.13 (95%CI: 2.59 to 3.78) Based on data from 1349 participants in 18 studies | Moderate† |
| JSB+ACEI/ARB vs. ACEI/ARB | Odds Ratio: 2.36 (95%CI: 1.41 to 3.95) Based on data from 103 participants in one study | Low†‡ |
| SYKF+ACEI/ARB vs. ACEI/ARB | Odds Ratio: 2.45 (95%CI: 1.69 to 3.55) Based on data from 262 participants in four studies | Low†‡ |
| TG+ACEI/ARB vs. ACEI/ARB | Odds Ratio: 2.81 (95%CI: 2.28 to 3.47) Based on data from 1260 participants in 20 studies | Moderate† |
| HBHG+ACEI/ARB vs. BL+ACEI/ARB | Odds Ratio: 0.76 (95%CI: 0.42 to 1.38) Based on indirect evidence | Very low†‡* |
| HK+ACEI/ARB vs. BL+ACEI/ARB | Odds Ratio: 0.96 (95%CI: 0.63 to 1.45) Based on indirect evidence | Very low†‡* |
| JSB+ACEI/ARB vs. BL+ACEI/ARB | Odds Ratio: 0.72 (95%CI: 0.37 to 1.41) Based on indirect evidence | Very low†‡* |
| SYKF+ACEI/ARB vs. BL+ACEI/ARB | Odds Ratio: 0.75 (95%CI: 0.42 to 1.33) Based on indirect evidence | Very low†‡* |
| TG+ACEI/ARB vs. BL+ACEI/ARB | Odds Ratio: 0.86 (95%CI: 0.56 to 1.32) Based on indirect evidence | Very low†‡* |
| HK+ACEI/ARB vs. HBHG+ACEI/ARB | Odds Ratio: 1.26 (95%CI: 0.78 to 2.03) Based on indirect evidence | Very low†‡* |
| JSB+ACEI/ARB vs. HBHG+ACEI/ARB | Odds Ratio: 0.95 (95%CI: 0.50 to 1.81) Based on indirect evidence | Very low†‡* |
| SYKF+ACEI/ARB vs. HBHG+ACEI/ARB | Odds Ratio: 0.98 (95%CI: 0.54 to 1.79) Based on indirect evidence | Very low†‡* |
| TG+ACEI/ARB vs. HBHG+ACEI/ARB | Odds Ratio: 1.13 (95%CI: 0.70 to 1.82) Based on indirect evidence | Very low†‡* |
| JSB+ACEI/ARB vs. HK+ACEI/ARB | Odds Ratio: 0.75 (95%CI: 0.44 to 1.29) Based on indirect evidence | Very low†‡* |
| SYKF+ACEI/ARB vs. HK+ACEI/ARB | Odds Ratio: 0.78 (95%CI: 0.51 to 1.20) Based on indirect evidence | Very low†‡* |
| TG+ACEI/ARB vs. HK+ACEI/ARB | Odds Ratio: 0.9 (95%CI: 0.67 to 1.20) Based on indirect evidence | Very low†‡* |
| SYKF+ACEI/ARB vs. JSB+ACEI/ARB | Odds Ratio: 1.04 (95%CI: 0.52 to 2.09) Based on indirect evidence | Very low†‡* |
| TG+ACEI/ARB vs. JSB+ACEI/ARB | Odds Ratio: 1.19 (95%CI: 0.70 to 2.03) Based on indirect evidence | Very low†‡* |
| TG+ACEI/ARB vs. SYKF+ACEI/ARB | Odds Ratio: 1.15 (95%CI: 0.76 to 1.73) Based on indirect evidence | Very low†‡* |

†Rated down 1 level for risk of bias.

‡Rated down 1 level for imprecision.

*Rated down 1 level for Indirectness.

6.2. GRADE summary of findings for 24-hour urinary protein quantity for different comparisons

| Comparations | Study results and measurements | Certainty in effect estimates |
| --- | --- | --- |
|  |  |  |
| BL+ACEI/ARB vs. ACEI/ARB | Mean Difference: -0.83  (95%CI: -1.21 to -0.44)  Based on data from 538 participants in seven studies | Moderate† |
| HBHG+ACEI/ARB vs. ACEI/ARB | Mean Difference: -0.56  (95%CI: -1.07 to -0.05)  Based on data from 134 participants in two studies | Low†‡ |
| HK+ACEI/ARB vs. ACEI/ARB | Mean Difference: -0.71  (95%CI: -0.87 to -0.55)  Based on data from 1370 participants in 19 studies | Moderate† |
| JSB+ACEI/ARB vs. ACEI/ARB | Mean Difference: -0.48  (95%CI: -0.94 to -0.03)  Based on data from 265 participants in three studies | Low†‡ |
| SYKF+ACEI/ARB vs. ACEI/ARB | Mean Difference: -0.65  (95%CI: -1.02 to -0.28)  Based on data from 355 participants in six studies | Moderate |
| TG+ACEI/ARB vs. ACEI/ARB | Mean Difference: -0.50  (95%CI: -0.65 to -0.36)  Based on data from 967 participants in 15 studies | Moderate† |
| HBHG+ACEI/ARB vs. BL+ACEI/ARB | Mean Difference: 0.27  (95%CI: -0.36 to 0.90)  Based on indirect evidence | Very low†‡* |
| HK+ACEI/ARB vs. BL+ACEI/ARB | Mean Difference: 0.12  (95%CI: -0.29 to 0.53)  Based on indirect evidence | Very low†‡* |
| JSB+ACEI/ARB vs. BL+ACEI/ARB | Mean Difference: 0.35  (95%CI: -0.18 to 0.88)  Based on indirect evidence | Very low†‡* |
| SYKF+ACEI/ARB vs. BL+ACEI/ARB | Mean Difference: 0.18  (95%CI: -0.27 to 0.63)  Based on indirect evidence | Very low†‡* |
| TG+ACEI/ARB vs. BL+ACEI/ARB | Mean Difference: 0.33  (95%CI: -0.02 to 0.68)  Based on indirect evidence | Very low†‡* |
| HK+ACEI/ARB vs. HBHG+ACEI/ARB | Mean Difference: -0.15  (95%CI: -0.72 to 0.42)  Based on indirect evidence | Very low†‡* |
| JSB+ACEI/ARB vs. HBHG+ACEI/ARB | Mean Difference: 0.08  (95%CI: -0.57 to 0.73)  Based on indirect evidence | Very low†‡* |
| SYKF+ACEI/ARB vs. HBHG+ACEI/ARB | Mean Difference: -0.09  (95%CI: -0.70 to 0.52)  Based on indirect evidence | Very low†‡* |
| TG+ACEI/ARB vs. HBHG+ACEI/ARB | Mean Difference: 0.06  (95%CI: -0.51 to 0.63)  Based on indirect evidence | Very low†‡* |
| JSB+ACEI/ARB vs. HK+ACEI/ARB | Mean Difference: 0.23  (95%CI: -0.28 to 0.74)  Based on indirect evidence | Very low†‡* |
| SYKF+ACEI/ARB vs. HK+ACEI/ARB | Mean Difference: 0.06  (95%CI: -0.38 to 0.50)  Based on indirect evidence | Very low†‡* |
| TG+ACEI/ARB vs. HK+ACEI/ARB | Mean Difference: 0.21  (95%CI: -0.04 to 0.46)  Based on indirect evidence | Very low†‡* |
| SYKF+ACEI/ARB vs. JSB+ACEI/ARB | Mean Difference: -0.17  (95%CI: -0.78 to 0.44)  Based on indirect evidence | Very low†‡* |
| TG+ACEI/ARB vs. JSB+ACEI/ARB | Mean Difference: -0.02  (95%CI: -0.59 to 0.55)  Based on indirect evidence | Very low†‡* |
| TG+ACEI/ARB vs. SYKF+ACEI/ARB | Mean Difference: 0.15  (95%CI: -0.32 to 0.62)  Based on indirect evidence | Very low†‡* |

†Rated down 1 level for risk of bias.

‡Rated down 1 level for imprecision.

*Rated down 1 level for Indirectness.

6.3. GRADE summary of findings for serum creatinine for different comparisons

| Comparations | Measurements | Certainty in effect estimates |
| --- | --- | --- |
|  |  |  |
| BL+ACEI/ARB vs. ACEI/ARB | Mean Difference: -14.29 (95%CI: -17.36 to -11.14)  Based on data from 376 participants in five studies | Moderate† |
| HBHG+ACEI/ARB vs. ACEI/ARB | Mean Difference: -26.63 (95%CI: -47.91 to -5.35)  Based on data from 234 participants in three studies | Low†‡ |
| HK+ACEI/ARB vs. ACEI/ARB | Mean Difference: -6.53 (95%CI: -10.26 to -2.80)  Based on data from 817 participants in 11 studies | Low†‡ |
| JSB+ACEI/ARB vs. ACEI/ARB | Mean Difference: -8.03  (95%CI: -18.51 to 2.45)  Based on data from 163 participants in two studies | Low†‡ |
| SYKF+ACEI/ARB vs. ACEI/ARB | Mean Difference: -13.10 (95%CI: -21.84 to -4.36)  Based on data from 315 participants in five studies | Low†‡ |
| TG+ACEI/ARB vs. ACEI/ARB | Mean Difference: -10.71 (95%CI: -13.41 to -8.01)  Based on data from 962 participants in 14 studies | Low†‡ |
| HBHG+ACEI/ARB vs. BL+ACEI/ARB | Mean Difference: -12.38 (95%CI: -33.66 to 8.90)  Based on indirect evidence | Very low†‡* |
| HK+ACEI/ARB vs. BL+ACEI/ARB | Mean Difference: 7.72 (95%CI: 3.99 to 11.45)  Based on indirect evidence | Very low†‡* |
| JSB+ACEI/ARB vs. BL+ACEI/ARB | Mean Difference: 6.22 (95%CI: -4.26 to 16.70)  Based on indirect evidence | Very low†‡* |
| SYKF+ACEI/ARB vs. BL+ACEI/ARB | Mean Difference: 1.15 (95%CI: -7.59 to 9.89)  Based on indirect evidence | Very low†‡* |
| TG+ACEI/ARB vs. BL+ACEI/ARB | Mean Difference: 3.54 (95%CI: 0.84 to 6.24)  Based on indirect evidence | Very low†‡* |
| HK+ACEI/ARB vs. HBHG+ACEI/ARB | Mean Difference: 17.30  (95%CI: -25.03 to 59.63)  Based on indirect evidence | Very low†‡* |
| JSB+ACEI/ARB vs. HBHG+ACEI/ARB | Mean Difference: 18.60 (95%CI: 8.12 to 29.08)  Based on indirect evidence | Very low†‡* |
| SYKF+ACEI/ARB vs. HBHG+ACEI/ARB | Mean Difference: 13.53  (95%CI: 4.79 to 22.27)  Based on indirect evidence | Very low†‡* |
| TG+ACEI/ARB vs. HBHG+ACEI/ARB | Mean Difference: 15.92 (95%CI: 13.22 to 18.62)  Based on indirect evidence | Very low†‡* |
| JSB+ACEI/ARB vs. HK+ACEI/ARB | Mean Difference: -1.50 (95%CI: -11.98 to 8.98)  Based on indirect evidence | Very low†‡* |
| SYKF+ACEI/ARB vs. HK+ACEI/ARB | Mean Difference: -6.57  (95%CI: -15.31 to 2.17)  Based on indirect evidence | Very low†‡* |
| TG+ACEI/ARB vs. HK+ACEI/ARB | Mean Difference: -4.18 (95%CI: -6.88 to -1.48)  Based on indirect evidence | Very low†‡* |
| SYKF+ACEI/ARB vs. JSB+ACEI/ARB | Mean Difference: -5.07  (95%CI: -15.55 to 5.41)  Based on indirect evidence | Very low†‡* |
| TG+ACEI/ARB vs. JSB+ACEI/ARB | Mean Difference: -2.68 (95%CI: -5.38 to 0.02)  Based on indirect evidence | Very low†‡* |
| TG+ACEI/ARB vs. SYKF+ACEI/ARB | Mean Difference: 2.39 (95%CI: -0.31 to 5.09)  Based on indirect evidence | Very low†‡* |

†Rated down 1 level for risk of bias.

‡Rated down 1 level for imprecision.

*Rated down 1 level for Indirectness.

6.4. GRADE summary of findings for blood urea nitrogen for different comparisons

| Comparations | Measurements | Certainty in effect estimates |
| --- | --- | --- |
|  |  |  |
| BL+ACEI/ARB vs. ACEI/ARB | Mean Difference: -1.77  (95%CI: -3.45 to -0.09)  Based on data from 190 participants in three studies | Low†‡ |
| HBHG+ACEI/ARB vs. ACEI/ARB | Mean Difference: -0.31  (95%CI: -1.29 to 0.67)  Based on data from 172 participants in two studies | Low†‡ |
| HK+ACEI/ARB vs. ACEI/ARB | Mean Difference: -0.53  (95%CI: -1.11 to 0.05)  Based on data from 482 participants in six studies | Moderate† |
| SYKF+ACEI/ARB vs. ACEI/ARB | Mean Difference: -0.74  (95%CI: -1.98 to 0.50)  Based on data from 170 participants in two studies | Low†‡ |
| TG+ACEI/ARB vs. ACEI/ARB | Mean Difference: -2.53  (95%CI: -4.54 to -0.52)  Based on data from 534 participants in seven studies | Moderate† |
| HBHG+ACEI/ARB vs. BL+ACEI/ARB | Mean Difference: 1.46  (95%CI: -0.52 to 3.44)  Based on indirect evidence | Very low†‡* |
| HK+ACEI/ARB vs. BL+ACEI/ARB | Mean Difference: 1.24  (95%CI: -0.54 to 3.02)  Based on indirect evidence | Very low†‡* |
| SYKF+ACEI/ARB vs. BL+ACEI/ARB | Mean Difference: 1.03  (95%CI: -1.00 to 3.06)  Based on indirect evidence | Very low†‡* |
| TG+ACEI/ARB vs. BL+ACEI/ARB | Mean Difference: -0.76  (95%CI: -2.94 to 1.42)  Based on indirect evidence | Very low†‡* |
| HK+ACEI/ARB vs. HBHG+ACEI/ARB | Mean Difference: -0.22  (95%CI: -1.38 to 0.94)  Based on indirect evidence | Very low†‡* |
| SYKF+ACEI/ARB vs. HBHG+ACEI/ARB | Mean Difference: -0.43  (95%CI: -1.86 to 1.00)  Based on indirect evidence | Very low†‡* |
| TG+ACEI/ARB vs. HBHG+ACEI/ARB | Mean Difference: -2.22  (95%CI: -4.40 to -0.04)  Based on indirect evidence | Very low†‡* |
| SYKF+ACEI/ARB vs. HK+ACEI/ARB | Mean Difference: -0.21  (95%CI: -1.55 to 1.13)  Based on indirect evidence | Very low†‡* |
| TG+ACEI/ARB vs. HK+ACEI/ARB | Mean Difference: -2.00  (95%CI: -4.10 to 0.10)  Based on indirect evidence | Very low†‡* |
| TG+ACEI/ARB vs. SYKF+ACEI/ARB | Mean Difference: -1.79  (95%CI: -4.10 to 0.52)  Based on indirect evidence | Very low†‡* |

†Rated down 1 level for risk of bias.

‡Rated down 1 level for imprecision.

*Rated down 1 level for Indirectness.

6.5. GRADE summary of findings for hemoglobin for different comparisons

| Comparations | Measurements | Certainty in effect estimates |
| --- | --- | --- |
|  |  |  |
| BL+ACEI/ARB vs. ACEI/ARB | Mean Difference: 9.41  (95%CI: 8.18 to 10.64)  Based on data from 186 participants in two studies | Low†‡ |
| HBHG+ACEI/ARB vs. ACEI/ARB | Mean Difference: 3.80  (95%CI: 0.42 to 7.18)  Based on data from 62 participants in one study | Very low†‡‡ |
| HK+ACEI/ARB vs. ACEI/ARB | Mean Difference: 2.45  (95%CI: 1.55 to 3.35)  Based on data from 848 participants in 12 studies | Moderate† |
| JSB+ACEI/ARB vs. ACEI/ARB | Mean Difference: 4.45  (95%CI: 2.69 to 6.21)  Based on data from 163 participants in two studies | Low†‡ |
| SYKF+ACEI/ARB vs. ACEI/ARB | Mean Difference: 7.18  (95%CI: 5.13 to 9.23)  Based on data from 102 participants in two studies | Low†‡ |
| TG+ACEI/ARB vs. ACEI/ARB | Mean Difference: 3.88  (95%CI: 2.65 to 5.11)  Based on data from 633 participants in ten studies | Moderate |
| HBHG+ACEI/ARB vs. BL+ACEI/ARB | Mean Difference: -5.61  (95%CI: -9.12 to -2.10)  Based on indirect evidence | Very low†‡* |
| HK+ACEI/ARB vs. BL+ACEI/ARB | Mean Difference: -6.96  (95%CI: -8.22 to -5.70)  Based on indirect evidence | Very low†‡* |
| JSB+ACEI/ARB vs. BL+ACEI/ARB | Mean Difference: -4.96  (95%CI: -7.06 to -2.86)  Based on indirect evidence | Very low†‡* |
| SYKF+ACEI/ARB vs. BL+ACEI/ARB | Mean Difference: -2.23  (95%CI: -4.69 to 0.23)  Based on indirect evidence | Very low†‡* |
| TG+ACEI/ARB vs. BL+ACEI/ARB | Mean Difference: -5.53  (95%CI: -7.10 to -3.96)  Based on indirect evidence | Very low†‡* |
| HK+ACEI/ARB vs. HBHG+ACEI/ARB | Mean Difference: -1.35  (95%CI: -4.90 to 2.20)  Based on indirect evidence | Very low†‡* |
| JSB+ACEI/ARB vs. HBHG+ACEI/ARB | Mean Difference: 0.65  (95%CI: -3.27 to 4.57)  Based on indirect evidence | Very low†‡* |
| SYKF+ACEI/ARB vs. HBHG+ACEI/ARB | Mean Difference: 3.38  (95%CI: -0.59 to 7.35)  Based on indirect evidence | Very low†‡* |
| TG+ACEI/ARB vs. HBHG+ACEI/ARB | Mean Difference: 0.08  (95%CI: -3.42 to 3.58)  Based on indirect evidence | Very low†‡* |
| JSB+ACEI/ARB vs. HK+ACEI/ARB | Mean Difference: 2.00  (95%CI: -0.16 to 4.16)  Based on indirect evidence | Very low†‡* |
| SYKF+ACEI/ARB vs. HK+ACEI/ARB | Mean Difference: 4.73  (95%CI: 2.42 to 7.04)  Based on indirect evidence | Very low†‡* |
| TG+ACEI/ARB vs. HK+ACEI/ARB | Mean Difference: 1.43  (95%CI: -0.07 to 2.93)  Based on indirect evidence | Very low†‡* |
| SYKF+ACEI/ARB vs. JSB+ACEI/ARB | Mean Difference: 2.73  (95%CI: -0.11 to 5.57)  Based on indirect evidence | Very low†‡* |
| TG+ACEI/ARB vs. JSB+ACEI/ARB | Mean Difference: -0.57  (95%CI: -2.73 to 1.59)  Based on indirect evidence | Very low†‡* |
| TG+ACEI/ARB vs. SYKF+ACEI/ARB | Mean Difference: -3.30  (95%CI: -5.59 to -1.01)  Based on indirect evidence | Very low†‡* |

†Rated down 1 level for risk of bias.

‡Rated down 1 level for imprecision.

‡‡Rated down 2 levels for imprecision.

*Rated down 1 level for Indirectness.

6.6. GRADE summary of findings for adverse drug reactions for different comparisons

| Comparations | Measurements | Certainty in effect estimates |
| --- | --- | --- |
|  |  |  |
| BL+ACEI/ARB vs. ACEI/ARB | Odds Ratio: 1.02  (95%CI: 0.43 to 2.41)  Based on data from 146 participants in two studies | Low†‡ |
| HBHG+ACEI/ARB vs. ACEI/ARB | Odds Ratio: 1.10  (95%CI: 0.34 to 3.54)  Based on data from 62 participants in one study | Low†‡ |
| HK+ACEI/ARB vs. ACEI/ARB | Odds Ratio: 1.25  (95%CI: 0.89 to 1.76)  Based on data from 783 participants in ten studies | Moderate† |
| JSB+ACEI/ARB vs. ACEI/ARB | Odds Ratio: 2.00  (95%CI: 0.17 to 23.43)  Based on data from 60 participants in one study | Very low†‡‡ |
| TG+ACEI/ARB vs. ACEI/ARB | Odds Ratio: 0.80  (95%CI: 0.57 to 1.12)  Based on data from 1008 participants in 15 studies | Moderate† |
| HBHG+ACEI/ARB vs. BL+ACEI/ARB | Odds Ratio: 1.08  (95%CI: 0.25 to 4.67)  Based on indirect evidence | Very low†‡* |
| HK+ACEI/ARB vs. BL+ACEI/ARB | Odds Ratio: 1.23  (95%CI: 0.42 to 3.60)  Based on indirect evidence | Very low†‡* |
| JSB+ACEI/ARB vs. BL+ACEI/ARB | Odds Ratio: 1.96  (95%CI: 0.15 to 25.32)  Based on indirect evidence | Very low†‡* |
| TG+ACEI/ARB vs. BL+ACEI/ARB | Odds Ratio: 0.78  (95%CI: 0.26 to 2.37)  Based on indirect evidence | Very low†‡* |
| HK+ACEI/ARB vs. HBHG+ACEI/ARB | Odds Ratio: 1.14  (95%CI: 0.30 to 4.29)  Based on indirect evidence | Very low†‡* |
| JSB+ACEI/ARB vs. HBHG+ACEI/ARB | Odds Ratio: 1.82  (95%CI: 0.12 to 27.04)  Based on indirect evidence | Very low†‡* |
| TG+ACEI/ARB vs. HBHG+ACEI/ARB | Odds Ratio: 0.73  (95%CI: 0.18 to 2.93)  Based on indirect evidence | Very low†‡* |
| JSB+ACEI/ARB vs. HK+ACEI/ARB | Odds Ratio: 1.60  (95%CI: 0.12 to 21.57)  Based on indirect evidence | Very low†‡* |
| TG+ACEI/ARB vs. HK+ACEI/ARB | Odds Ratio: 0.64  (95%CI: 0.41 to 1.00)  Based on indirect evidence | Very low†‡* |
| TG+ACEI/ARB vs. JSB+ACEI/ARB | Odds Ratio: 0.40  (95%CI: 0.03 to 5.42)  Based on indirect evidence | Very low†‡* |

†Rated down 1 level for risk of bias.

‡Rated down 1 level for imprecision.

‡‡Rated down 1 level for imprecision.

*Rated down 1 level for Indirectness.

Appendix 7. SUCRA plots

7.1 SUCRA plot for overall response rate


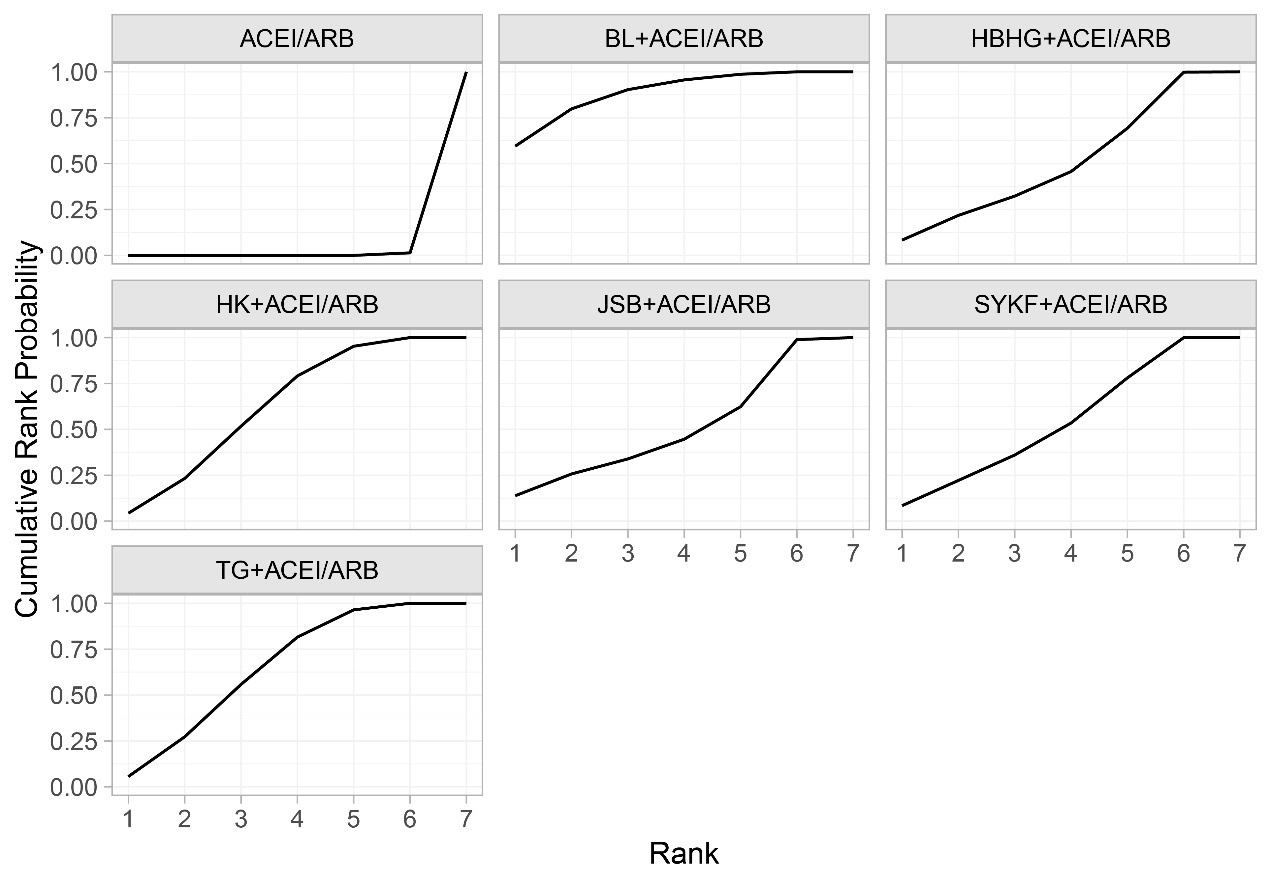


7.2 SUCRA plot for 24-hour urinary protein quantity


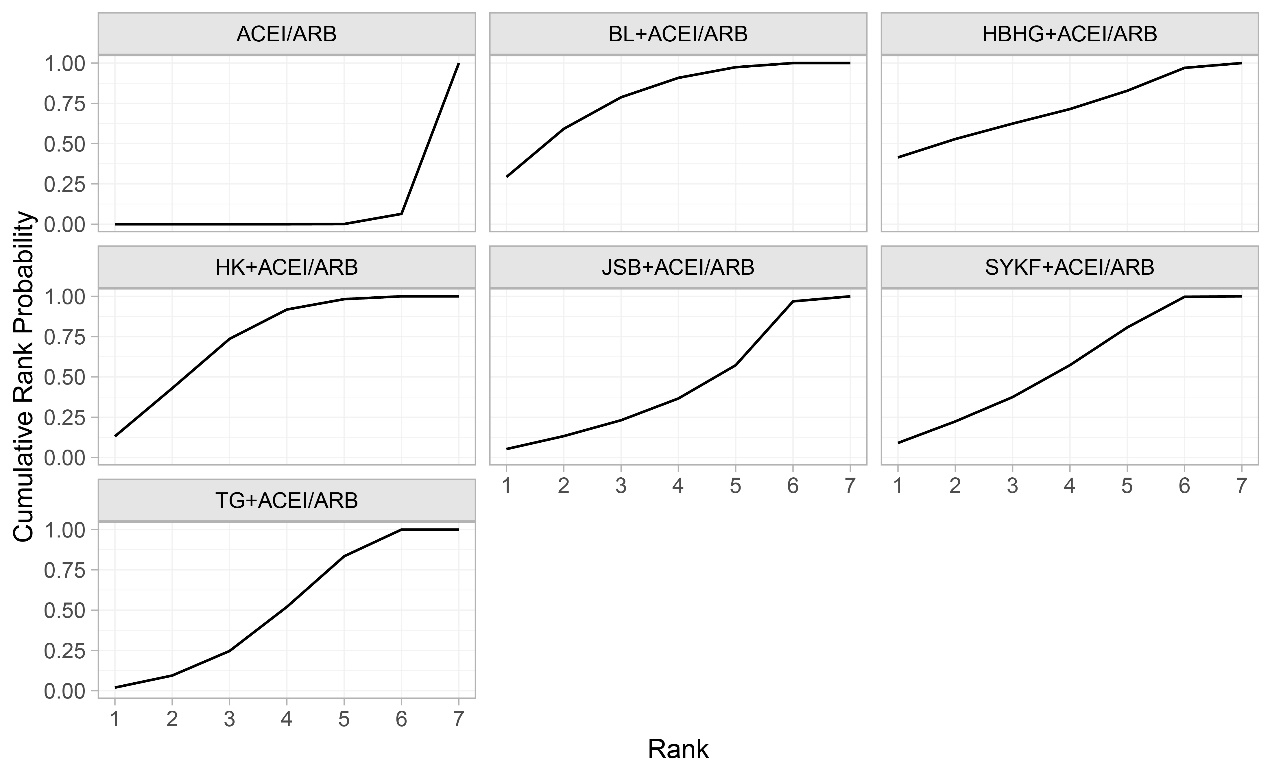


7.3 SUCRA plot for serum creatinine


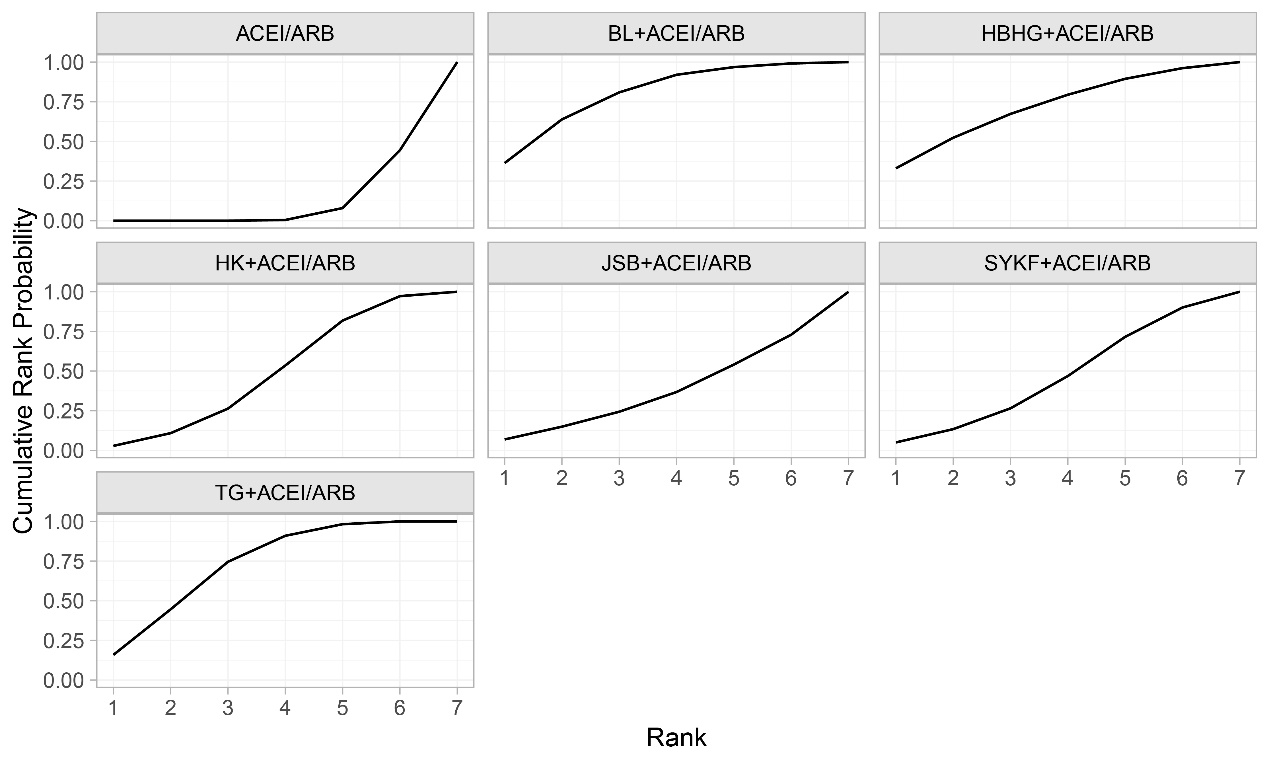


7.4 SUCRA plot for blood urea nitrogen


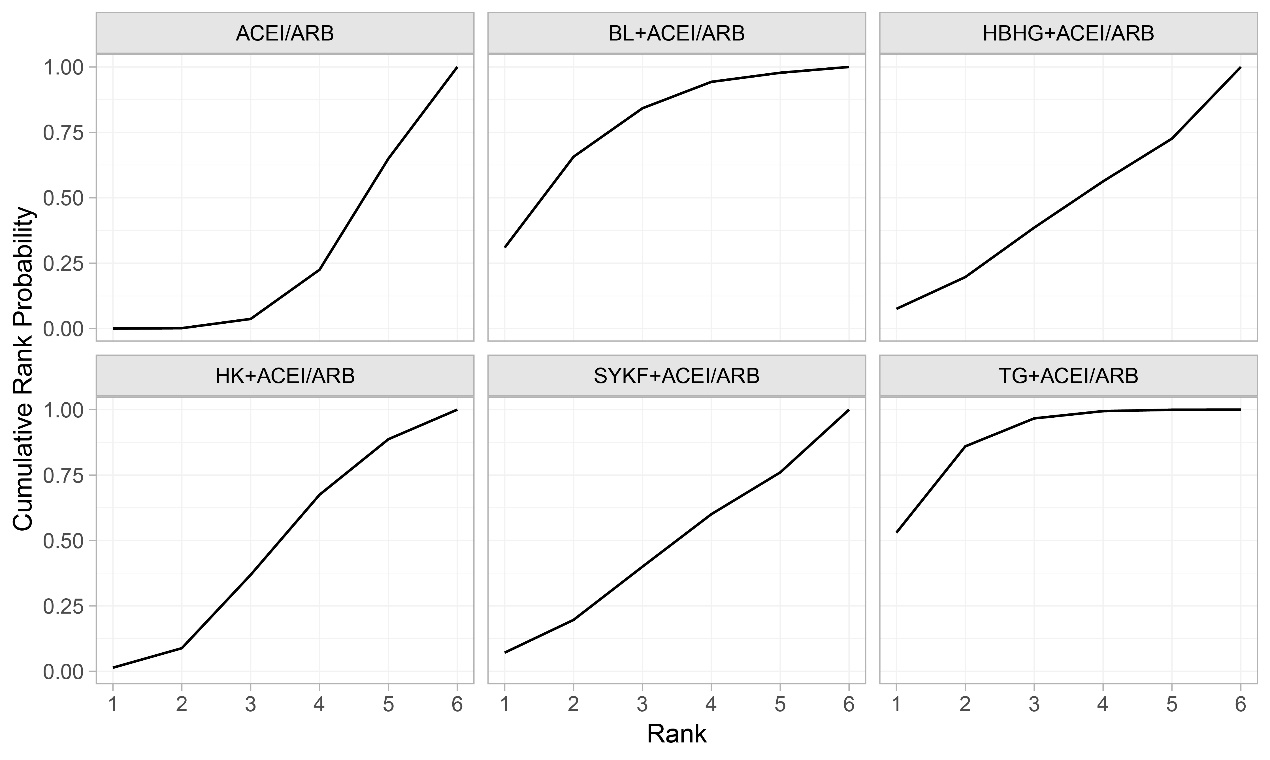


7.5 SUCRA plot for hemoglobin


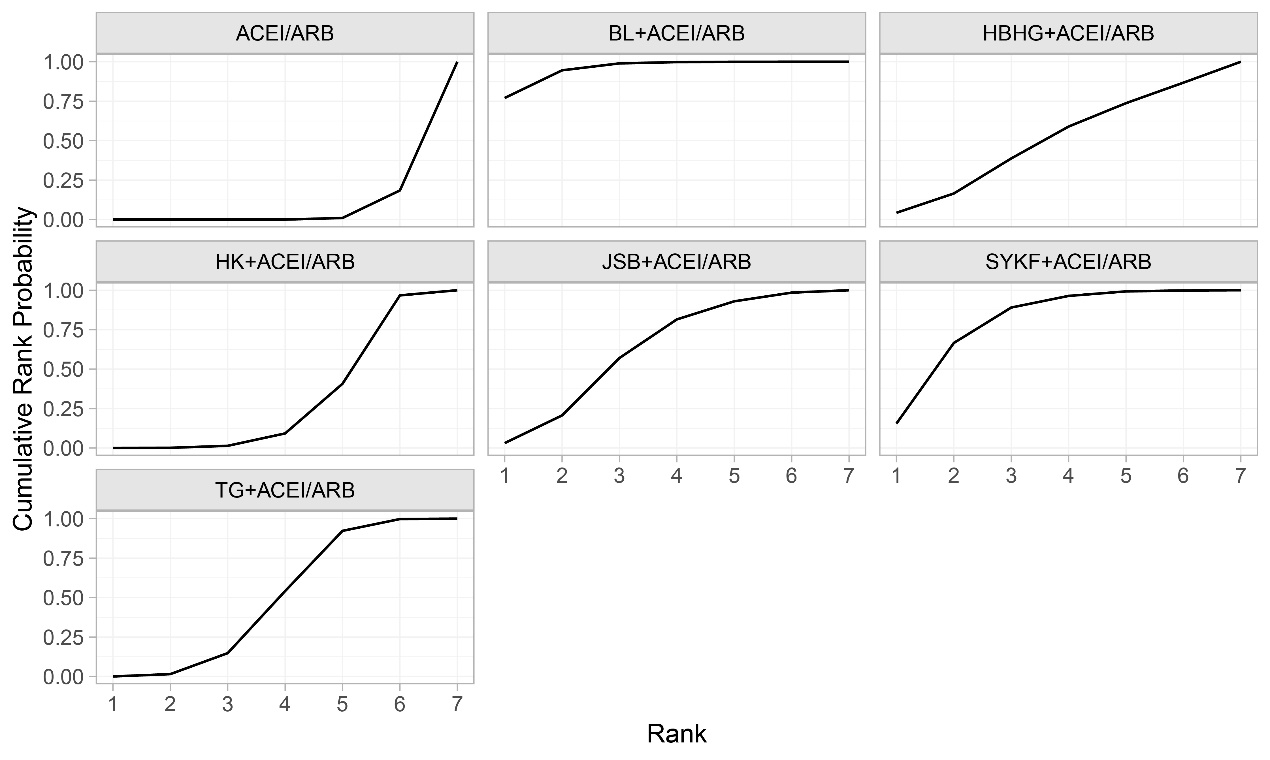


7.6 SUCRA plot for adverse drug reactions


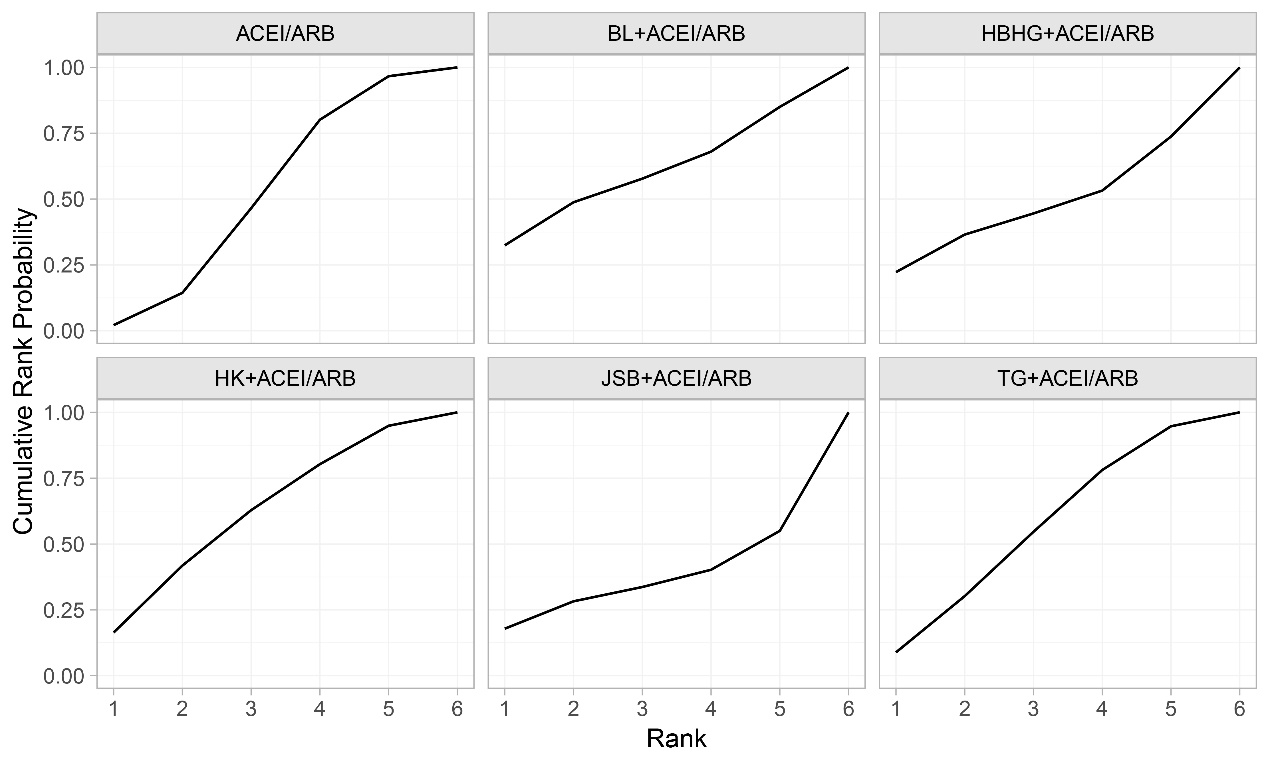


Appendix 8. Assessment of SUCRA

| Outcome | Treatment | SUCRA |
| --- | --- | --- |
| Overall response rate | BL+ACEI/ARB | 0.87 |
|  | TG+ACEI/ARB | 0.61 |
|  | HK+ACEI/ARB | 0.59 |
|  | SYKF+ACEI/ARB | 0.50 |
|  | JSB+ACEI/ARB | 0.47 |
|  | HBHG+ACEI/ARB | 0.46 |
|  | ACEI/ARB | 0.00 |
| 24-hour urinary protein quantity | BL+ACEI/ARB | 0.77 |
|  | HK+ACEI/ARB | 0.69 |
|  | HBHG+ACEI/ARB | 0.68 |
|  | SYKF+ACEI/ARB | 0.51 |
|  | TG+ACEI/ARB | 0.45 |
|  | JSB+ACEI/ARB | 0.39 |
|  | ACEI/ARB | 0.01 |
| Serum creatinine | BL+ACEI/ARB | 0.78 |
|  | TG+ACEI/ARB | 0.71 |
|  | HBHG+ACEI/ARB | 0.70 |
|  | HK+ACEI/ARB | 0.45 |
|  | SYKF+ACEI/ARB | 0.42 |
|  | JSB+ACEI/ARB | 0.35 |
|  | ACEI/ARB | 0.09 |
| Blood urea nitrogen | TG+ACEI/ARB | 0.87 |
|  | BL+ACEI/ARB | 0.74 |
|  | HK+ACEI/ARB | 0.41 |
|  | SYKF+ACEI/ARB | 0.41 |
|  | HBHG+ACEI/ARB | 0.39 |
|  | ACEI/ARB | 0.18 |
| Hemoglobin | BL+ACEI/ARB | 0.95 |
|  | SYKF+ACEI/ARB | 0.78 |
|  | JSB+ACEI/ARB | 0.59 |
|  | HBHG+ACEI/ARB | 0.47 |
|  | TG+ACEI/ARB | 0.44 |
|  | HK+ACEI/ARB | 0.25 |
|  | ACEI/ARB | 0.03 |
| Adverse drug reactions | HK+ACEI/ARB | 0.59 |
|  | BL+ACEI/ARB | 0.58 |
|  | TG+ACEI/ARB | 0.53 |
|  | ACEI/ARB | 0.48 |
|  | HBHG+ACEI/ARB | 0.46 |
|  | JSB+ACEI/ARB | 0.35 |

Appendix 9. Detailed Adverse Drug Reactions by Intervention

| Intervention | Total patients | Nausea | Vomiting | Abdominal distension | Diarrhea | Dizziness | Headache | ALT elevation | Leukopenia | Amenorrhea/menstrual disorder | Skin rash | Other ADRs | Total ADRs | ADR rate (%) |
| --- | --- | --- | --- | --- | --- | --- | --- | --- | --- | --- | --- | --- | --- | --- |
| BL + ACEI/ARB | 81 | 1 | 0 | 0 | 1 | 0 | 1 | 0 | 0 | 0 | 0 | 1 (hypertension) | 4 | 4.94 |
| JSB + ACEI/ARB | 30 | 3 | 0 | 0 | 0 | 0 | 0 | 0 | 0 | 0 | 0 | 0 | 3 | 10 |
| HBHG + ACEI/ARB | 32 | 3 | 0 | 0 | 0 | 0 | 0 | 0 | 0 | 0 | 0 | 4 (anorexia, fatigue) | 7 | 21.88 |
| TG + ACEI/ARB | 468 | 12 | 2 | 10 | 0 | 3 | 1 | 3 | 2 | 3 | 2 | 10 (fatigue, infection, hyperglycemia) | 48 | 10.26 |
| HK + ACEI/ARB | 395 | 21 | 0 | 2 | 1 | 3 | 0 | 1 | 0 | 0 | 0 | 11 (dry mouth, elevated creatinine) | 39 | 9.87 |
| ACEI/ARB alone | 653 | 32 | 1 | 5 | 0 | 5 | 1 | 2 | 0 | 0 | 1 | 31 (cough, Cushing's syndrome, osteoporosis) | 77 | 11.79 |

Notes: ACEI, angiotensin-converting enzyme inhibitor; ARB, angiotensin receptor blocker; BL, Bailing capsule; HBHG, Huobahuagen Tablet; HK, Huangkui capsule; TG, Tripterygium glycosides; JSB, Jinshuibao capsule.

Appendix 10. The results of sensitivity analysis

10.1 The sensitivity analysis result of overall response rate


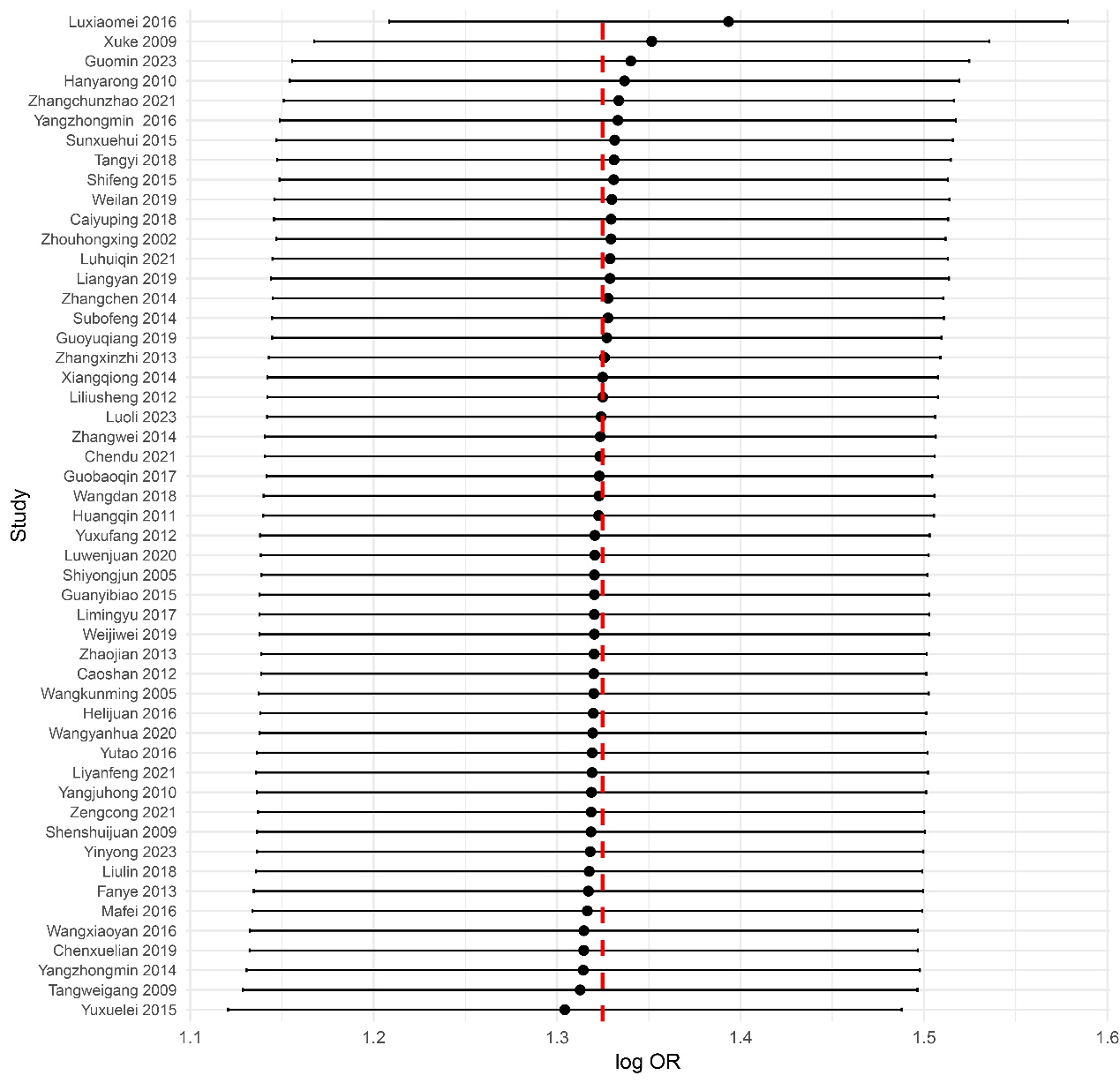


10.2 The sensitivity analysis result of 24-hour urinary protein quantity


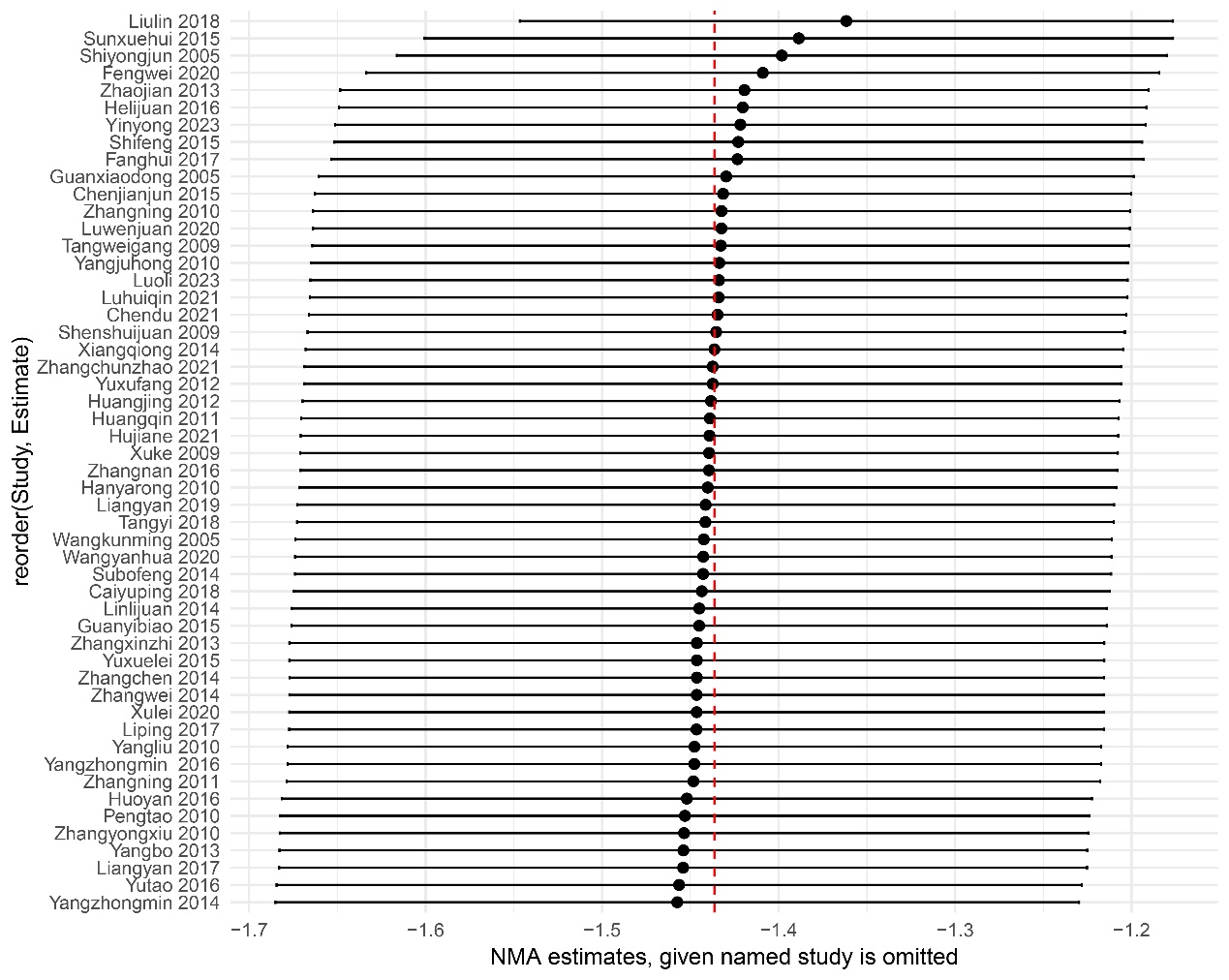


10.3 The sensitivity analysis result of serum creatinine


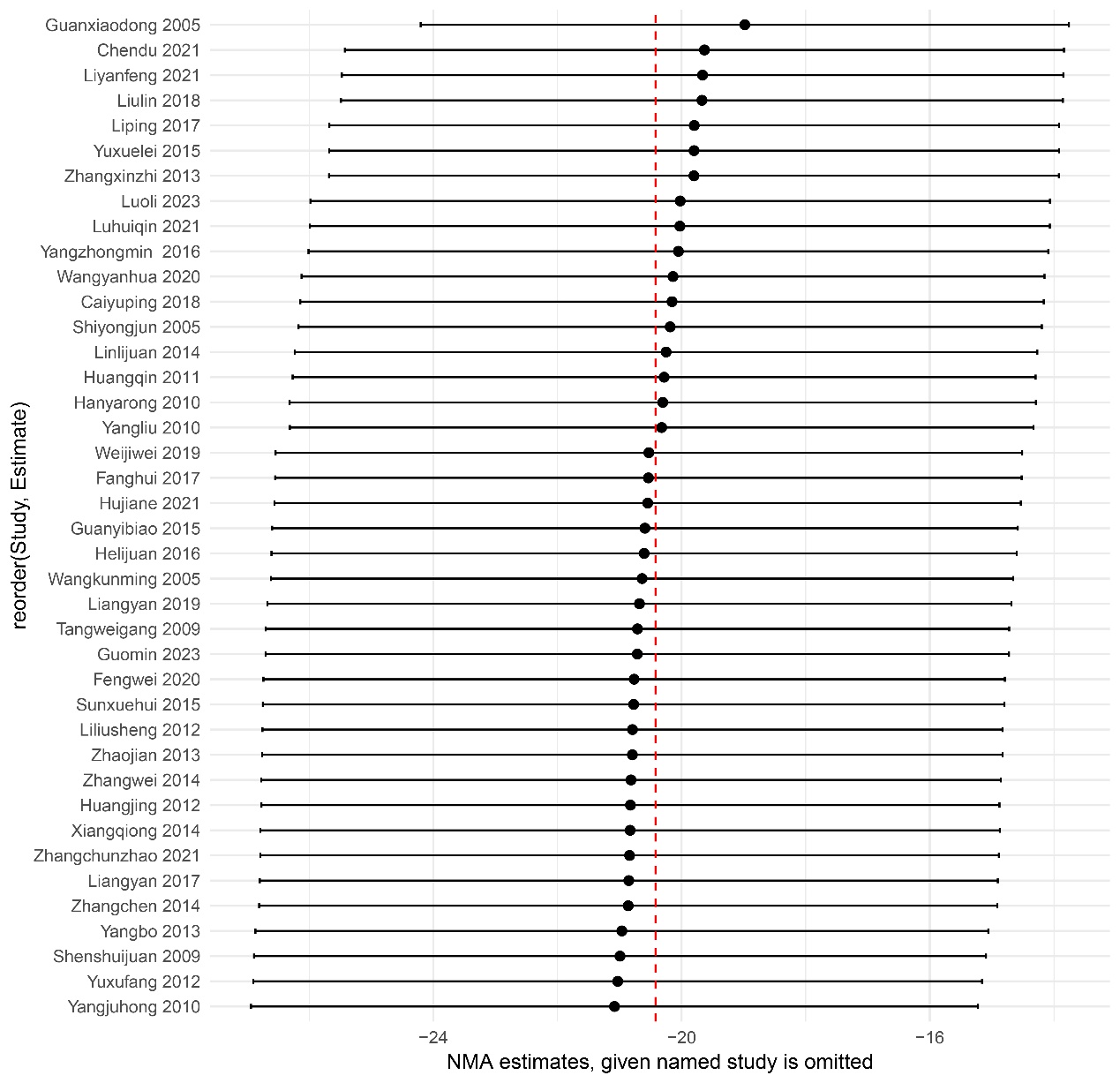


10.4 The sensitivity analysis result of blood urea nitrogen


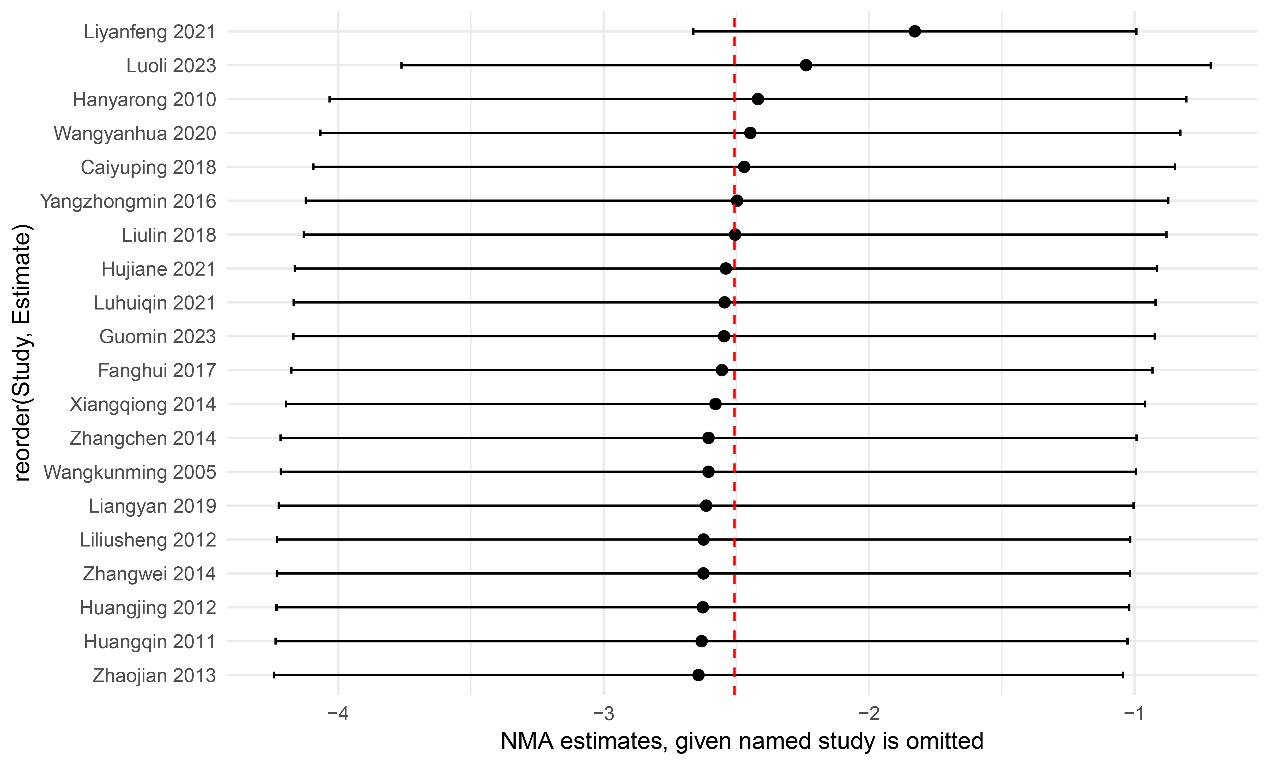


10.5 The sensitivity analysis result of hemoglobin


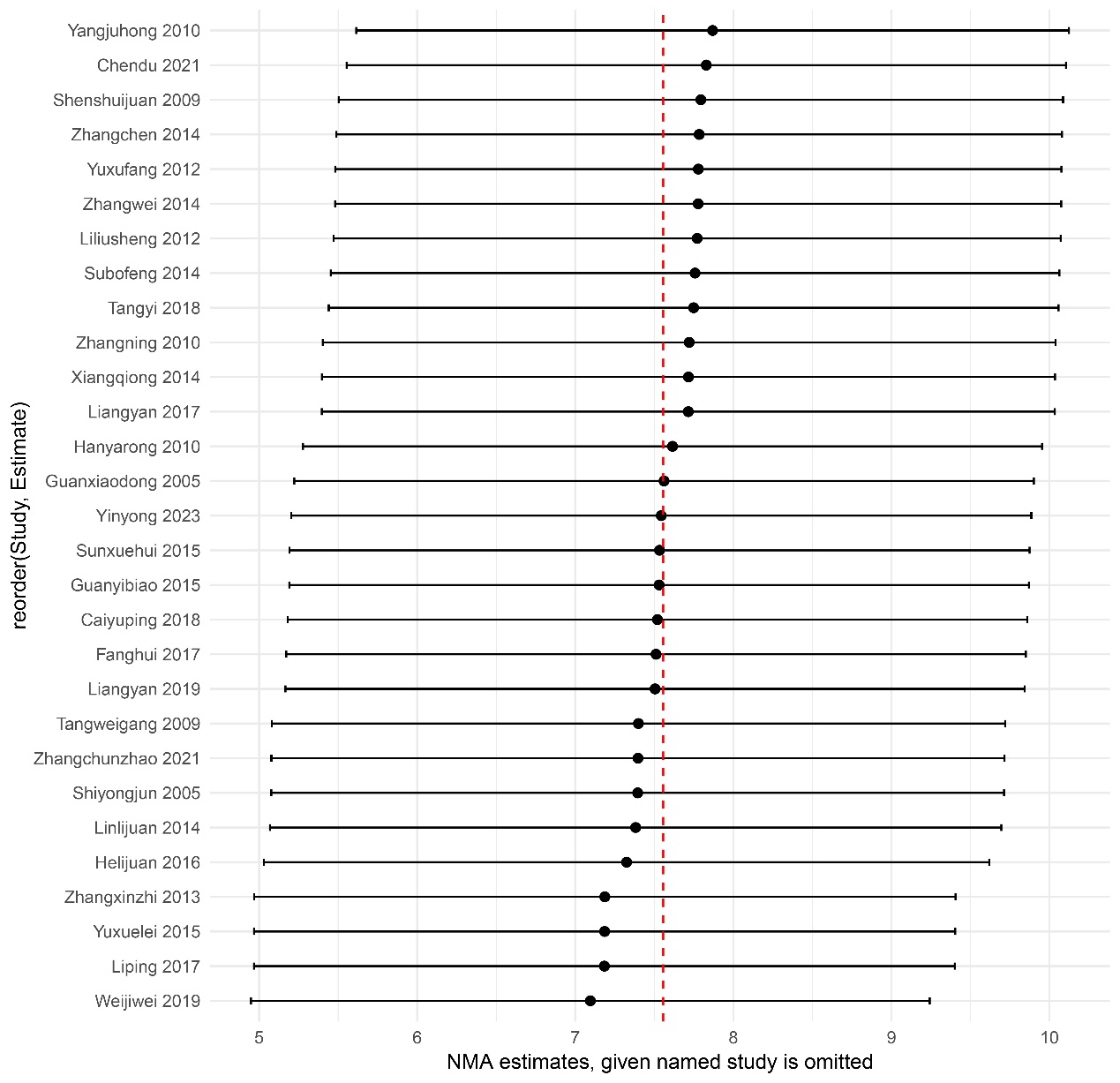


10.6 The sensitivity analysis result of adverse drug reactions


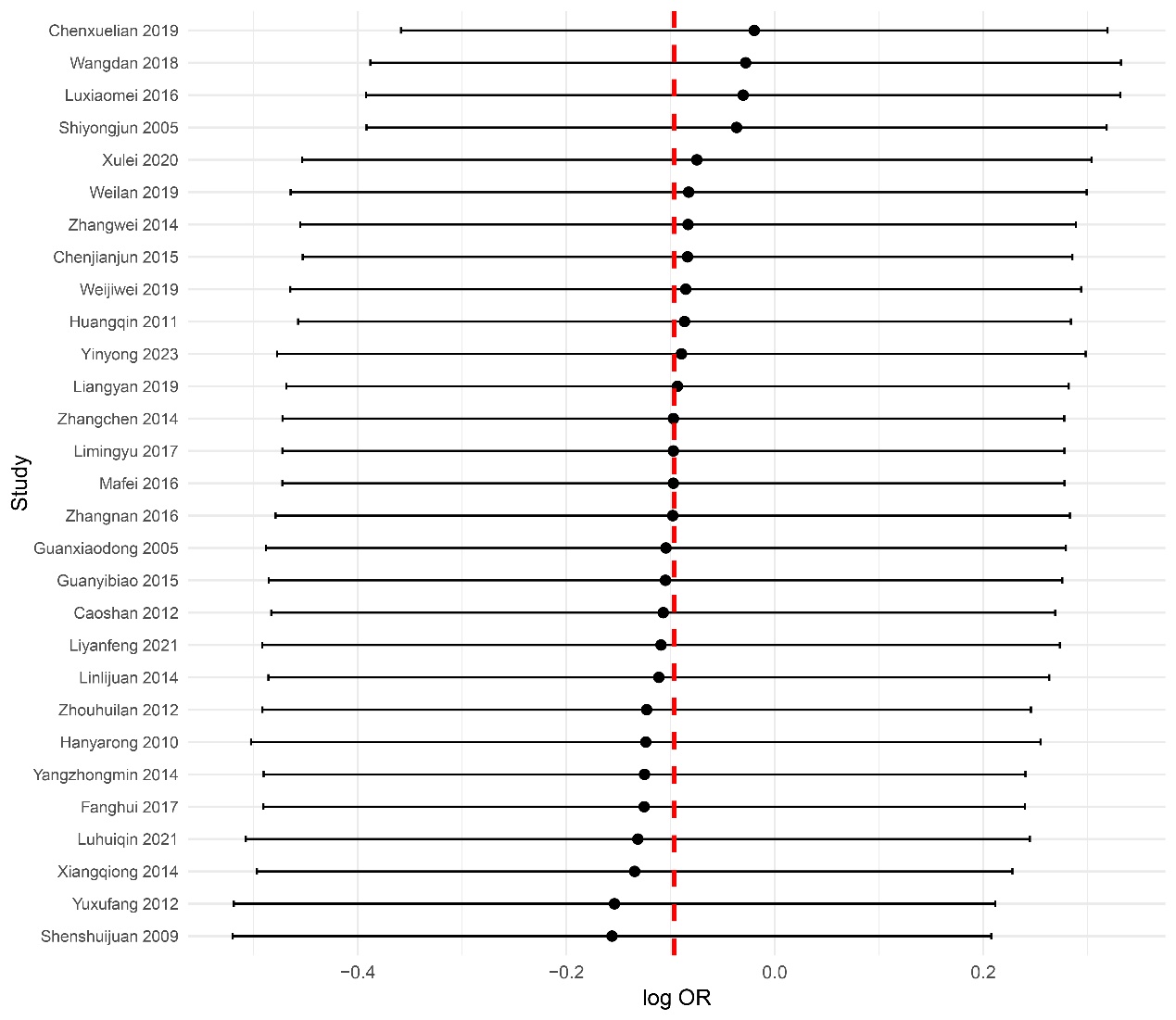

Supplement: Supplementary file 1 [file Supplementaryfile1.docx]
